# The Sterol Transporter Npc2c Controls Intestinal Stem Cell Mitosis and Host–Microbiome Interactions in *Drosophila*

**DOI:** 10.3390/metabo13101084

**Published:** 2023-10-16

**Authors:** Constantina Neophytou, Euripides Soteriou, Chrysoula Pitsouli

**Affiliations:** Department of Biological Sciences, University of Cyprus, 1 University Avenue, 2109 Aglantzia, Cyprus; kneofy02@ucy.ac.cy (C.N.); esoter01@ucy.ac.cy (E.S.)

**Keywords:** *Npc2*, cholesterol, ecdysone, dysbiosis

## Abstract

Cholesterol is necessary for all cells to function. The intracellular cholesterol transporters Npc1 and Npc2 control sterol trafficking and their malfunction leads to Neimann–Pick Type C disease, a rare disorder affecting the nervous system and the intestine. Unlike humans that encode single Npc1 and Npc2 transporters, flies encompass two Npc1 (Npc1a-1b) and eight Npc2 (Npc2a-2h) members, and most of the *Npc2* family genes remain unexplored. Here, we focus on the intestinal function of *Npc2c* in the adult. We find that *Npc2c* is necessary for intestinal stem cell (ISC) mitosis, maintenance of the ISC lineage, survival upon pathogenic infection, as well as tumor growth. Impaired mitosis of *Npc2c*-silenced midguts is accompanied by reduced expression of *Cyclin* genes, and genes encoding ISC regulators, such as *Delta*, *unpaired1* and *Socs36E*. ISC-specific *Npc2c* silencing induces *Attacin*-*A* expression, a phenotype reminiscent of Gram-negative bacteria overabundance. Metagenomic analysis of *Npc2c*-depleted midguts indicates intestinal dysbiosis, whereby decreased commensal complexity is accompanied by increased gamma-proteobacteria. ISC-specific *Npc2c* silencing also results in increased cholesterol aggregation. Interestingly, administration of the non-steroidal ecdysone receptor agonist, RH5849, rescues mitosis of *Npc2c*-silenced midguts and increases expression of the ecdysone response gene *Broad*, underscoring the role of *Npc2c* and sterols in ecdysone signaling. Assessment of additional *Npc2* family members indicates potential redundant roles with *Npc2c* in ISC control and response to ecdysone signaling. Our results highlight a previously unidentified essential role of *Npc2c* in ISC mitosis, as well as an important role in ecdysone signaling and microbiome composition in the *Drosophila* midgut.

## 1. Introduction

Proliferating cells rely on nutrients acquired from the environment and directed into metabolic pathways to support cell-specific functions and maintain homeostasis [1]. One of the most widely studied nutrients is cholesterol, which is essential for all multicellular organisms: it is a steroid hormone precursor [2], it is involved in the integrity and fluidity of the lipid bilayer [3] and contributes to cell signaling [4]. Maintenance of cholesterol levels under specific limits is necessary for normal cellular functions, whereas plasma cholesterol deficiency due to impaired cholesterol metabolism may lead to serious malformations and behavioral or developmental problems [5]. A cholesterol-rich diet has been associated with increased risk for cardiovascular disease, obesity and gastrointestinal cancer [6,7,8,9,10]. Since the intestinal stem cells (ISCs) are considered the cells of origin of intestinal tumors, and evidence from research in *Drosophila* shows that gut microbiota-derived nutrients can modulate ISCs [11], one could speculate that cholesterol availability and metabolism may impinge on ISC function and, therefore, contribute to tumorigenesis. Indeed, a recent study in mice has shown that dietary cholesterol acts as a mitogen for ISCs and increases cholesterol synthesis, promoting tumorigenesis [12]. Similarly, another study in *Drosophila melanogaster* has shown that dietary cholesterol alters the Notch signaling pathway, a key ISC differentiation pathway, the dysfunction of which leads to tumors [13].

In mammals, the mechanisms regulating cholesterol biosynthesis are well characterized [14], but those controlling its uptake from the diet, removal and turnover have only recently started to be understood. *Drosophila* cannot synthesize cholesterol [15], but it has been used extensively to study its homeostasis because the mechanisms for cholesterol uptake and intracellular trafficking are conserved between mammals and flies [16]. For example, *Drosophila* encodes several genes involved in the absorption, metabolism and transport of sterols [17,18,19,20]. Cholesterol is absorbed through receptor-mediated endocytosis by intestinal enterocytes (ECs) and it is subsequently transported to lysosomes. Then, under the control of the Niemann–Pick type C (Npc) proteins, it moves to organelles, such as the endoplasmic reticulum, and upon esterification, it is transported to peripheral tissues [21]. Cholesterol homeostasis is achieved through the sterol-regulatory element binding proteins (SREBPs) and liver X receptors (LXRs). SREBPs are transcription factors [22], and LXRs are nuclear receptors mediating transcriptional responses upon cholesterol binding [23]. Unlike mammals, the single *Drosophila* SREBP ortholog responds to palmitate instead of sterols [22,24]. On the other hand, the *Drosophila* nuclear hormone receptor 96 (DHR96), a homolog of the mammalian LXRs [20,25], is crucial for cholesterol homeostasis. In particular, at low cholesterol levels, DHR96 is active and targets genes, including NPC gene family members, whereas at high cholesterol, DHR96 is inhibited [20]. The second homologue of the mammalian LXRs in flies is the Ecdysone receptor (EcR), which binds with high affinity to the *Drosophila* steroid hormone, ecdysone [26]. *Drosophila* uses cholesterol as a precursor to synthesize ecdysone, which is converted into the metabolically active 20-hydroxy-ecdysone (20E) by the P450 enzyme [27]. Similar to vertebrates, where steroids are needed for growth and reproduction, in flies, 20E regulates developmental transitions, such as larval molting and metamorphosis, acts on nervous and reproductive systems, and affects lifespan and metabolism [28,29]. In the fly intestine, it has been shown to induce proliferation and gut growth [30], as well as stem cell fate decisions [31].

The *Drosophila* midgut is maintained by pluripotent ISCs, which typically divide asymmetrically to self-renew and produce two types of progenitor cells, the enteroblasts (EBs) or the enteroendocrine precursors (pre-EEs). EBs and pre-EEs terminally differentiate into the absorptive ECs and enteroendocrine cells (EEs), respectively. ISC proliferation and differentiation are regulated by conserved signaling pathways including Notch, Jak/Stat, EGFR, Insulin-receptor, Wnt/Wingless, Hippo and Dpp-signaling during homeostasis and regeneration [32]. In addition, systemic hormones secreted in the hemolymph act on distant organs and may regulate ISC proliferation [33]. Interestingly, recent evidence suggests that mating-induced 20E produced in the ovaries activates the EcR in neighboring posterior midgut progenitors (ISCs and EBs), inducing the expression of the early response gene, *Ecdysone*-*induced*-*protein*-*75B* (*Eip75B*). *Eip75B*, the human PPARg and REV-ERB homologue [34,35], promotes EB differentiation towards the EC fate upon mating [31]. Similarly, 20E feeding or mating-produced 20E induces ISC mitosis through progenitor-specific upregulation of two EcR target genes, *Eip75B* and the transcription factor *Broad* (*Br*) [30]. Taken together, previous findings indicate a significant role of ecdysone and sterol metabolism in ISC-mediated intestinal homeostasis.

In humans, *Npc1* and *Npc2* mutations cause Neimann–Pick Type C disease [36], a rare disorder characterized by impaired cholesterol trafficking, which affects the central nervous system, bone marrow, liver, spleen, and intestine [37,38]. In addition, a role of *Npc2* in olfactory sensing through regulation of putative odorant carrier proteins has been identified in mites [39,40], while *Npc1* mutant mice exhibit olfactory impairment due to astrocytosis and microgliosis [41]. The human *Npc1* and *Npc2* genes act synergistically to maintain homeostatic cholesterol metabolism through trafficking between late endosomes/lysosomes [36,42,43]. In flies, the *Npc* gene family encodes known regulators of cholesterol metabolism and ecdysteroid biosynthesis [44]. Humans have only two *Npc* genes, *Npc1* and *Npc2*, whereas the *Drosophila* genome encodes two *Npc1* homologs (*Npc1a* and *Npc1b*) and 8 *Npc2* homologs (*Npc2a*-*h*) [18]. *Npc1a* controls intracellular sterol trafficking needed for ecdysteroid biosynthesis [21,45] and *Npc1b* is involved in sterol uptake in the midgut [46]. Among the eight Npc2 proteins, Npc2a has the highest amino acid similarity to the human Npc2 (36% identity) [18]. Npc2a and Npc2b have redundant roles in intracellular cholesterol trafficking and ecdysteroid biosynthesis [13]. Among the fly *Npc* genes, *Npc1b* and *Npc2c*-*e* are regulated by DHR96 and dietary cholesterol [20]. In addition, transcriptome analysis of *DHR96* mutant adult males found *Npc1b*, *Npc2c* and *Npc2e* significantly upregulated, and *Npc2d*, *Npc2g* and *Npc2h* downregulated compared to controls [47]. However, the roles of *Npc2c*-*h* remain a mystery. Npc2 proteins, are subdivided in three groups based on the number of cysteine residues they encompass. *Npc2a*-*c* and *Npc2f* contain six, *Npc2d*-*e* contain seven and *Npc2g*-*h* contain eight cysteine residues [48]. Representative members of each group, the Npc2a, Npc2e and Npc2h, were found to bind to conserved microbial components, such as bacterial lipopolysaccharide (LPS), peptidoglycan (PG), lipoteichoic acid (LTA) and the lipid A in vitro. In S2 cells, Npc2a and Npc2e binding on PG activates *Diptericin* (*Dipt*), which encodes an antimicrobial peptide (AMP) that activates the immune deficiency (IMD) pathway, suggesting innate immunity roles for Npc2 proteins in *Drosophila* [48]. This finding highlighted their similarity to vertebrate Npc2, which also regulates immune signaling, via binding on LPS and lipid A.

Here, to begin to understand the unexplored roles of Npc2 family genes in *Drosophila*, we investigated the function of *Npc2c* in the adult *Drosophila* midgut. We found that *Npc2c* is necessary in the adult intestine for ISC mitosis, homeostasis and tumorigenesis. Npc2c exerts its effects on physiology through the regulation of intestinal sterol quantity and the ecdysone pathway.

## 2. Materials and Methods

### 2.1. Drosophila Stocks and Rearing

All stocks were routinely maintained at 18 °C or 25 °C on a 12:12 h light/dark cycle on a standard fly medium, 10 g agar, 43.5 g cornmeal, 30 g yeast, 24 g sugar, 5.3 mL of a 20% Tegasept dissolved in 100% ethanol and 3.8 mL 99% propionic acid for 1000 mL. The following Gal4 lines were used for cell-type specific expression: for progenitors (ISCs and EBs), *w*; *esg*-*Gal4 UAS*-*GFP tub*-*Gal80^ts^* (*esg^ts^*) [49]; for ISCs, *w*; *esg*-*Gal4 UAS*-*GFP*; *Su*(*H*)-*Gal80 tub*-*Gal80^ts^* (*ISC^ts^*) [50]; for EBs, *w*; *Su*(*H*)-*Gal4 UAS*-*CD8GFP tub*-*Gal80^ts20^/CyO* (*Su*(*H*)*^ts^*) [51]; and for ECs, *w tub*-*Gal80^ts^/FM7; Myo1A*-*Gal4 UAS*- *EGFP/CyO* (*MyO^ts^*) [52].

Other lines used in this study were *w^1118^* (BDSC# 6326), *w*; *esg*-*Gal4 UAS*-*eGFP tub*-*Gal80^ts^/CyO*; *UAS*-*Ras1^Q13^/TM6C* [53]. All the *UAS*-*RNAi* lines used in this study are listed in Table S1 (Key Resources Table). For MARCM analysis, the following genotypes were used: *w hs*-*FLP tub*-*Gal4 UAS*-*nlsGFP/FM7*; *FRT82B tub*-*Gal80/TM6B* [54], *w*; *FRT82B arm*-*lacZ/TM6B* (BDSC# 7369), *w*; *Npc2c^RNAi1^/CyO*; *FRT82B arm*-*lacZ* (this study).

GAL4-UAS crosses carrying *tub*-*Gal80^ts^* [55,56] were reared at 18 °C and female adult flies (5 to 7 days old) were transferred to 29 °C for 5 days for transgene induction.

To test the efficiency of *w*; *UAS*-*Npc2c^RNAi1^* and *w*; *UAS*-*Npc2c^RNAi2^*, the flies were crossed to *w*; *Act5C*-*Gal4 UAS*-*GFP/CyO* and reared at 25 °C. Late L3 GFP-positive larvae were collected for RNA extraction.

### 2.2. MARCM Clones

For MARCM clones [57] *w hs*-*FLP tub*-*Gal4 UAS*-*nlsGFP/FM7*; *FRT82B tub*-*Gal80/TM6B* flies were crossed to *w*; *FRT82B arm*-*lacZ/TM6B* (control) or *w*; *Npc2c^RNAi1^/CyO*; *FRT82B arm*-*lacZ/TM6C* (*Npc2c*-silenced). Crosses were incubated at 25 °C. 2- to 4-day old adult female progeny were heat-shocked at 37 °C for 60 min to induce FLP. Heat-shocked flies were subsequently transferred to fresh standard medium every two days and the midguts of at least 10 flies of each genotype were dissected, fixed and immunostained after 1, 3, 7, 12 and 14 days. Quantification of clone number and size was performed on whole midguts under a fluorescence microscope (Zeiss Axioscope A.1, Oberkochen, Germany).

### 2.3. Oral Administration of Bacteria

Female mated 5-to-7-day-old flies were used for all feeding assays. *Pseudomonas aeruginosa* (*P.a.*): *P.a.* feeding was performed as previously described [58]. The *P.a*. strain PA14 was maintained as LB-50% glycerol stock at −80 °C. A single colony of PA14 was grown at 37 °C in liquid LB to OD_600nm_ = 3, corresponding to 5 × 10^9^ bacteria/mL. Female mature flies of the desired genotype were starved for 5 h and added in groups of 10 per fly vial containing a cotton ball at the bottom soaked with 5 mL of 0.5 mL PA14 OD_600nm_ = 3, 1 mL 20% sucrose and 3.5 mL ddH_2_O. For uninfected control, 1 mL sucrose 20% and 4 mL dH_2_O was used. Flies were incubated for 48 h at 29 °C (for all experiments utilizing the Gal4-UAS, unless otherwise noted).

### 2.4. Oral Administration of Chemicals

EcR agonist, RH5849: RH5849 oral administration was performed as previously described [31]. Fly food was reheated in the microwave and for each vial needed, 2 mL of liquid fly food was mixed with 340 mM RH5849 (DrEhrenstorfer#DRE-C16813000). Specifically, for 1 mL of fly food, 5 uL of 20 ug/mL stock solution, diluted in methanol, was added and thoroughly mixed. As a control (mock), an equivalent amount of methanol was added to the fly food. Groups of 10–15 female mature flies of the desired genotype were transferred from 18 °C (on standard fly food) to RH5849/methanol-supplemented vials at 29 °C for 5 days to allow RNAi induction. Flies were transferred on freshly prepared supplemented food, every day. Then, the flies were incubated for 48 h at 29 °C on sucrose 4% or *P.a.* supplemented with 340 mM RH5849 or methanol (control).

Cholesterol: *P.a.* infection assays with 250 μg/mL cholesterol were performed using cholesterol from a 25 mg/mL stock solution dissolved in 100% ethanol. Specifically, cotton balls at the bottom of the vials were each soaked with 0.5 mL *P.a.* OD_600nm_ = 3, 1 mL 20% sucrose, 3.45 mL ddH2O, and 0.05 mL of cholesterol stock solution. For uninfected controls, 1 mL 20% sucrose, 3.95 mL ddH2O, and 0.05 mL of cholesterol stock solution were used.

20 Hydroxyecdysone (20E): 20E was diluted in 100% ethanol (stock 5 mg/mL). Flies were transferred in 50 mL closed falcon tubes, and a Whatman paper disc was placed on the outside surface of their perforated lid. The disc was covered with heat-killed yeast paste (0.3 g/mL) supplemented with 20E at 0.008 mg/g diluted in ethanol and it was sealed with parafilm to reduce evaporation. Flies were allowed to feed on the yeast paste through small holes on the lid of the tubes. *UAS*-*RNAi* was induced for 5 days at 29 °C. The flies were flipped daily on fresh tubes and 2 days’ feeding with sucrose or *P.a.* was performed. Mock samples: absolute ethanol was added instead of 20E, at the same final concentration.

### 2.5. Midgut Tumorigenesis

Tumors were induced in the midgut ISCs/EBs via the Gal4-UAS-Gal80^ts^ system. *w*; *esg*-*Gal4 UAS*-*eGFP tub*-*Gal80^ts^*; *UAS*-*Ras1^Q13^* flies express GFP and the activated form of Ras-GTPase, Ras^Q13^, in ISCs/EBs. To simultaneously downregulate *Npc2c* in the progenitor compartment of *Ras^Q13^* tumorous intestines, we crossed *w*; *esg*-*Gal4 UAS*-*eGFP tub*-*Gal80^ts^*; *UAS*-*Ras1^Q13^* to *w*; *UAS*-*Npc2c^RNAi1^* and *w^1118^* (control). Crosses were reared at 18 °C, and emerging adults were maintained at 18 °C to mature for 5–7 days. Then, mated adult females were transferred to 29 °C to induce the transgenes for 1 day followed by 2 days feeding on a 4% sucrose (uninfected) or *P.a.* infection medium.

### 2.6. Dissections and Immunohistochemistry

Dissections of adult midguts and immunohistochemistry were performed as previously described [58]. Flies were dissected on Sylgard (VWR) plates in 1× PBS (130 mM NaCI, 70 mM NA_2_HPO_4_, 30 mM NaH_2_PO_4_), fixed in 4% formaldehyde (Polysciences, Warrington, PA, USA) in 1× PBS for 20 min at RT, and rinsed three times with 1× PBS. Blocking was performed in PBT (1× PBS, 0.2% Triton-Χ, 0.5% BSA) at RT for at least 20 min. Primary antibodies were diluted in PBT and incubated overnight at 4 °C. Primary antibodies were washed 3 times at RT for 10 min in PT (1× PBS, 0.2% Triton X-100). Tissues were incubated in secondary antibodies diluted in PBT including DAPI (Sigma, St. Louis, MI, USA) for 1–2 h at RT with mild shaking. Samples were washed 3 times and mounted in Vectashield (Vector Laboratories, Newark, CA, USA). The primary antibodies were rabbit anti-pH3 (Millipore 1:4000), mouse anti-Prospero (DSHB 1:100), chicken anti-GFP (Invitrogen, Waltham, MA, USA 1:2000), rabbit anti-GFP (Invitrogen 1:3000), rabbit anti-cleaved Caspase-3 (Cell signaling #9661 1:400), and mouse anti-Armadillo (DSHB 1:100). Secondary antibodies conjugated to Alexa 488 and Alexa 555 (Invitrogen) were used at 1:1000. DAPI was used to stain DNA (Sigma 1:4000 of 10 mg/mL stock).

### 2.7. Npc2c Polyclonal Antibody

A fragment corresponding to amino acids 1-165 of *Drosophila* Npc2c protein (UniProt ID: Q9VH31) was cloned by Boster (https://www.bosterbio.com, accessed on 10 June 2023) in tagged vectors. Bacterially expressed aa1-165 of Npc2c was purified and used to immunize rabbits to generate polyclonal antibodies. These are available by Boster (Cat# DZ41252).

### 2.8. Filipin Staining

For Filipin staining of free sterols, dissected midguts were fixed for 20 min in 4% formaldehyde (Polysciences) and washed three times with 1× PBS. Midguts were stained with 50 μg/mL filipin (from 25 mg/mL stock in DMSO) in PBS for 45 min in the dark. After two washes with 1× PBS, samples were mounted in Vectashield (Vector) and imaged by confocal microscope.

### 2.9. Survival Assay

Adult flies in triplicates of ten 3-to-5-day-old female flies for each genotype were infected with *P. aeruginosa* PA14, as descripted above. Flies were shifted to 29 °C and the number of dead and alive flies was recorded every 24 h.

### 2.10. Smurf Assay/Gut Permeability

The Smurf assay [59] was performed in triplicates of 15 female flies in each vial. Flies were maintained at 18 °C and transgene expression was induced for 5 days or 14 days at 29 °C. The flies were starved in empty vials for 5 h and then transferred for 5 h, 16 h at 29 °C in vials with cotton balls soaked with 5 mL 4% sucrose with 0.5% *w*/*v* Bromophenol blue pH = 7. Also, upon 5 days induction at 29 °C, flies were fed on *P.a*. infection mix for 16 h and then were transferred for 5 h on cotton balls with Bromophenol blue. The Smurf phenotype was assessed under the stereoscope and flies whose body was blue throughout (from head to tail) were recorded as Smurfs, indicating that the epithelial integrity is lost and the dye escaped in the hemolymph.

### 2.11. RNA Isolation and RT-qPCR

Total RNA was extracted from 25 dissected adult midguts per genotype per condition in triplicates using Qiazol (Qiagen, Hilden, Germany) and dissolved in RNase-free water. A total of 800 ng of total RNA was freed from genomic DNA using the RQ1 RNase-Free DNase Kit (Promega, Alexandria, New South Wales). Reverse transcription was performed using 145.4 ng of the total DNase-treated RNA using the TaKaRa PrimeScript RT Master Mix Kit. qPCR reactions were performed using gene-specific primers, the sequences of which are provided in Table S2 with the amplification program shown in Table S3, using the Bio-Rad CFX Manager 3.1 program. Gene expression was normalized to the expression of two references genes, *RpL32* and *a*-*tub* using the 2-ΔΔCt method. At least 3 biological replicates were used to calculate the mean and standard deviation.

### 2.12. Bacterial Load

Three externally sterilized (by brief dipping into 100% ethanol and drying) flies from each genotype/condition were used to determine bacterial colony-forming units (CFUs). Flies were placed into 2 mL Eppendorf tubes containing 200 μL BHI agar (BHI broth supplemented with 1 mL 0.5% Hemin and 0.5 mL of 0.5% Vitamin K1) and a stainless-steel 5 mm bead (Qiagen), and were homogenized with the TissueLyser II (Qiagen) at 50 Hz for 10 min. Total microflora CFUs were estimated by serial dilutions, and plating of 100 μL on BHI plates, which are then incubated overnight at 37 °C. CFUs per fly were calculated by dividing the number of CFUs on the plates by three, considering the dilution used. CFUs were performed twice, in triplicate.

### 2.13. Microbiota Analysis

A. Culture-independent. Female flies were surface-sterilized in 100% ethanol prior to dissection in sterile 1× PBS for 2 min. Forceps and dissection plates were also sterilized in 100% ethanol prior to dissection. Two pools of 50 dissected midguts per genotype were placed into 2 mL Eppendorf tubes containing 700 μL of lysis buffer (Invitrogen #A29790) and a stainless-steel 5 mm bead (Qiagen), and were homogenized with the TissueLyser II (Qiagen) at 50 Hz for 5 min. Bacterial DNA was extracted using the PurelinkTM Microbiome DNA purification kit (Invitrogen). Bacterial DNA was eluted in 90 uL of elution buffer and used for 16S rRNA sequencing. Primers targeting the V3/V4 regions were used for 16S metagenomic sequencing library preparation. Illumina MiSeq paired-end (2 × 300 bp) sequencing was performed (Macrogen Europe B.V., Amsterdam, The Netherlands), followed by an Operational Taxonomic unit (OTU) analysis for Bacteria and Archaea using the NCBI 16S database (Macrogen Europe B.V.). The raw 16S rRNA gene sequencing data are available from the National Center for Biotechnology Information (NCBI) with Sequence Read Archive (SRA) accession number PRJNA804129.

B. Culture-dependent. Several pools of 3 female individuals (5–7 day old from 18 °C and 5 days at 29 °C) were surface-sterilized in 100% ethanol, homogenized in 200 μL BHI and plated on BHI agar, as described above. Based on colony morphological characteristics, two representative isolates were observed. Each colony was used to inoculate 50 μL of sterile DNase/RNase-free water and DNA was extracted by boiling for 6 min at 95 °C and then 10 min on ice. Total DNA was used for bacterial 16S rRNA amplification with 27F and 1492R primers, using the KAPA HiFi HotStart Ready Mix PCR Kit (Roche, Basel, Switzerland). The 16S colony PCR amplification program is described in Table S4. PCR products were detected on 1% agarose gel (with 0.2 μg/mL Ethidium bromide) by electrophoresis. PCR products were purified using the NucleoSpin Gel and PCR Clean up (Macherey-Nagel, Düren, Germany) and sequenced (Macrogen Europe B.V.). The NCBI BLAST sequence analysis tool was used to analyze the sequences.

### 2.14. Image Acquisition and Analysis

The number of pH3-positive cells and MARCM clones was counted under the fluorescent microscope (Zeiss Axioscope A.1) at 20× magnification along the whole midgut.

All the images shown are stacks of optical sections acquired using the Leica TCS SP2 DMIRE2 confocal microscope. Confocal images were captured at 40× magnification, zoom 1× (unless otherwise noted) and 1024 × 1024 format and produced as a maximum projection of 10–15 serial sections. Images to be compared were acquired with the exact same settings. For cell quantification, at least 10 images from two sequential 37.5 × 37.5 μm frames of posterior regions P4(R5) and P3(R4c) were analyzed in Image J. Different channels were separated and each cell population was measured independently. For comparisons among the genotypes, the ratio of the total number of each cell population over the total number of cells per frame was calculated. Midgut area was measured by manually highlighting the surface of each midgut in each frame, using the Freehand selection tool of the software. Similarly, for EE quantification in tumorous midguts, images from the same regions were used for calculating the ratio of EEs per total nuclei per frame.

Sterol measurements (Filipin staining) were acquired from confocal maximum projection images which were stacks of 3–4 sections of the posterior regions P3(R4c). Fast acquisition was necessary because the fluorescence signal of filipin photobleaches quickly. The surface area of each gut per frame was manually highlighted using Image J and the fluorescence (area integrated intensity) of each midgut was measured. To calculate the corrected total midgut fluorescence, for each image, the product of the area and the mean background fluorescence was subtracted from the integrated intensity measured initially.

The EC nucleus maximum cross-section area was measured as previously described [51]. Confocal images of posterior regions P4(R5) and P3(R4c) captured using the same parameters as above. According to confocal photo properties, the distance in pixels was converted to μm (it is known that the distance of 1024 pixels equals 375 μm). Single-channel images (DAPI staining) were analyzed by adjusting the threshold to produce 2-pixel intensities on the photo: black and white. A binary version of each image was generated and the type of measurement was specified to the Analyze particles option. To increase measurement accuracy, the infinity value was set to either 1 or 2, to exclude calculation of random speckles in the photo. Particles smaller than 23 μm^2^ and merged nuclei were also excluded from the analysis. Measurements of area corresponding to each numbered nucleus were plotted using Prism 9 and Student’s *t*-test was used for statistical analysis.

### 2.15. Statistical Analysis

The numerical results are presented as mean ± standard deviation of the mean. Statistical significance was evaluated using the two-tailed Student’s *t*-test when comparing the averages of two groups of values, with the minimum of 10 values each. Comparisons between the relative mRNA levels in different genotypes/conditions was performed with the minimum of 3 biological replicates for each. For CFUs, we used at least 6 biological replicates and significance was tested using the Mann–Whitney U Test (www.socscistatistics.com, accessed on 15 June 2023). For fly survival, the Kaplan–Meier method was performed, using the log-rank test (MedCalc: https://www.mdcalc.com, accessed on 10 June 2022). The Prism 9 (v.9.0.0) software was used to perform all tests except the U-test. The type of statistical test used for each experiment and the number of samples tested are indicated in the figure legends. *p*-values are indicated as ns, not significant, * 0.01 < *p* ≤ 0.05, ** 0.001 < *p* ≤ 0.01, *** 0.0001 < *p* ≤ 0.001, **** *p* ≤ 0.0001.

## 3. Results

### 3.1. Npc2c Is Expressed in the Midgut Epithelium and Affects Physiology

**Figure 1:**
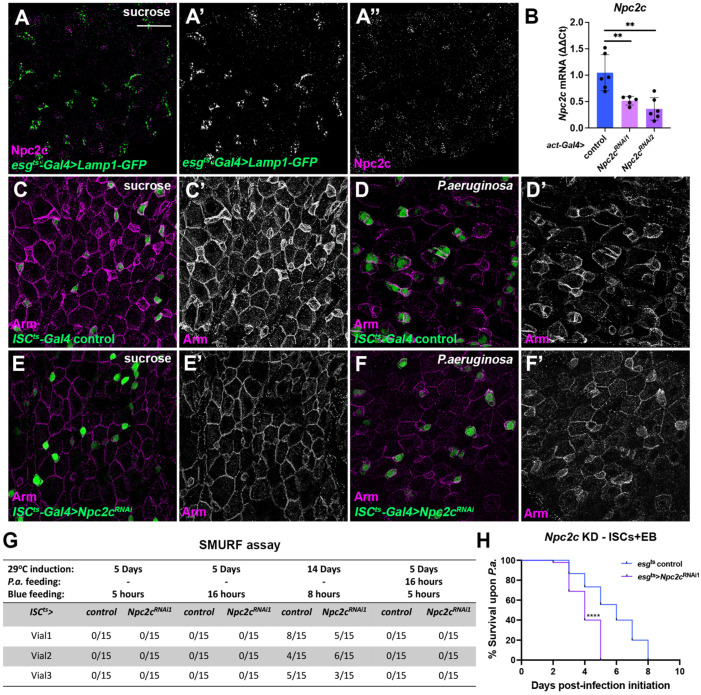
**Npc2c is expressed in intestinal progenitors and controls midgut cell morphology and organismal physiology.** (**A**) Npc2 protein expression (magenta) in the adult posterior midgut progenitors highlighted with *esg^ts^*-*Gal4* > *Lamp1*-*GFP* (green), which labels the lysosomes. (**A’**,**A”**) correspond to the separate green and magenta channels of (**A**), respectively. (**B**) *Npc2* mRNA expression in third instar larvae upon ubiquitous *Npc2c* knockdown with two independent *UAS*-*RNAi* lines (*UAS*-*Npc2c^RNAi1^* is *UAS*-*Npc2c^KK101583^* and *UAS*-*Npc2c^RNAi2^* is *UAS*-*Npc2c^GD31139^*) under the control of *Act5C*-*Gal4*. N = 6 biological replicates for each sample. (**C**–**F**) Adult posterior midguts of control *ISC^ts^*-*Gal4* (**C**,**D**) and *ISC^ts^*-*Gal4* > *Npc2c^RNAi1^* (**E**,**F**) without (**C**,**E**) and with *P.a.* infection (**D**,**F**) stained for Arm (magenta). GFP (green) labels the ISCs in all panels (**C**–**F**). Arm staining is shown separately in (**C’**–**F’**). (**G**) Smurf assay for *ISC^ts^*-*Gal4* control and *ISC^ts^*-*Gal4* > *Npc2c^RNAi1^* does not show any effect of *Npc2c* on gut permeability. (**H**) Adult *esg^ts^*-*Gal4* control and *esg^ts^*-*Gal4* > *Npc2c^RNAi1^* flies (triplicate experiment with 15 flies each) were subjected to continuous oral *P.a.* infection and their survival was measured. Scale bars in (**A**,**C**–**F**) are 48 μm. Error bars correspond to the standard deviation. Experiments were repeated at least twice. One-way ANOVA for multiple comparisons was used to test significance in (**B**); the Kaplan–Meier log rank test was used to assess significance in (**H**). ns, not significant; ** 0.001 < *p* ≤ 0.01, **** *p* ≤ 0.0001.

To start exploring the role of Npc2c, we assessed its expression in silico through database searches. According to the FlyAtlas2 [60], *Npc2c* expression is restricted to the larval and adult midgut. Cell type-specific adult midgut expression analysis [61] in FlygutSeq shows that the eight *Npc2* family members (*Npc2a*-*h*) exhibit variable expression in different intestinal cell populations (Figure S1A) and respond differently to pathogenic *Pseudomonas entomophila* (*P.e*.) infection (Figure S1B). In particular, *Npc2c* mRNA was detected at low levels in all types of intestinal cells and it was turned on specifically in ISC/EB progenitors upon *P.e.* infection. To assess Npc2c protein expression and localization in the adult midgut, we generated a polyclonal anti-Npc2c antibody (see Methods 2.7). We used the *esg^ts^*-*Gal4* (*esg*-*Gal4 UAS*-*EGFP tub*-*Gal80^ts^*) driver [49] to label ISCs and EBs of the posterior midgut with the lysosomal marker Lamp-GFP [62] and we assessed Npc2c expression using anti-Npc2c. We found that Npc2c was expressed in ICSs/EBs of the posterior midgut and exhibited vesicular localization, albeit rarely colocalizing with lysosomes (Figure 1A).

To assess the role of *Npc2c* in the adult intestinal epithelium, we used tissue-specific RNAi to silence the gene. First, we used RT-qPCR to test the knockdown efficiency of two independent *UAS*-*Npc2c^RNAi^* lines upon ubiquitous overexpression using *Act5C*-Gal4 in larvae. We found that both *UAS*-*Npc2c^RNAi1^* (VDRC KK101583) and *UAS*-*Npc2c^RNAi2^* (VDRC GD31139) led to significantly reduced Npc2c mRNA levels (0.51% for *Npc2c^RNAi1^* and 0.36% for *Npc2c^RNAi2^*) compared to controls at 25 °C (Figure 1B). Then, we silenced *Npc2c* specifically in the adult ISCs using the *ISC^ts^*-*Gal4* (*esg*-*Gal4 UAS*-*EGFP tub*-*Gal80^ts^ Su*(*H*)-*Gal80*) driver and noticed that *Npc2c* loss led to intestinal epithelial cell shape changes both in uninfected conditions and upon oral bacterial infection with *Pseudomonas aeruginosa* (*P.a.*), a pathogen causing intestinal regenerative inflammation [58]. Specifically, the *Npc2c*-silenced ISCs lost their triangular shape (Figure 1C–F). Immunostaining for the *Drosophila* β-catenin homologue, Armadillo (Arm), that decorates adherens junctions [63], confirmed that *Npc2*-deficient ISCs were morphologically different from wild-type because they lost membrane Arm and their shape became round (Figure 1C,E). Infected midguts exhibited inhomogeneous adherens junction staining, especially of the most apically positioned ECs, where Arm was significantly reduced (Figure 1D). The reduction of membrane Arm staining occurred also in all epithelial cells upon ISC-specific *Npc2c* knockdown (Figure 1F). Since Arm is deregulated upon ISC-specific *Npc2c* silencing, we hypothesized that intestinal epithelial integrity might be affected. To assess the latter, we performed Smurf assays in the presence and absence of *P.a.* at different time points (Figure 1G). Enumeration of Smurfs showed no difference between the control and *Npc2c*-deficient flies at different conditions (Figure 1G). Nevertheless, ISC/EB-specific *Npc2c* knockdown reduced the overall organismal fitness as it increased fly susceptibility to *P.a.* infection, underscored by the reduction of the lethal time 50% (LT50%) from >5 to 4 days compared to controls (Figure 1H). These results highlight that *Npc2c* expression in the adult intestinal progenitors impinges on fly physiology.

### 3.2. Npc2c Autonomously Controls ISC Mitosis

**Figure 2:**
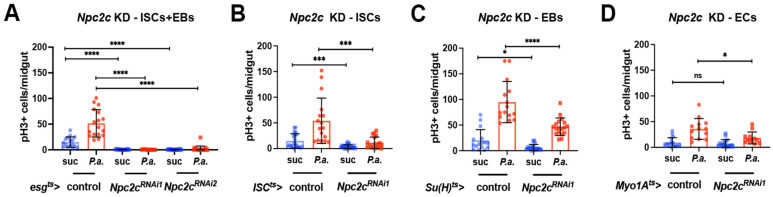
***Npc2c* is necessary in ISCs for midgut mitosis.** (**A**–**D**) Quantification of the mitotic index (pH3-positive cells per midgut) of control and *Npc2c*-silenced midguts in ISC/EB progenitors (**A**), ISCs (**B**), EBs (**C**) and ECs (**D**) without (suc, sucrose; blue bars) and with *P.a.* oral infection (red bars). Experiments were repeated at least twice. At least 15 midguts were used for each condition. Error bars correspond to the standard deviation. Statistical significance was tested with Student’s *t*-test in (**B**–**D**) and one-way ANOVA for multiple comparisons in (**A**) (N ≥ 12 per condition). ns, not significant; * 0.01 < *p* ≤ 0.05, *** 0.0001 < *p* ≤ 0.001, **** *p* ≤ 0.0001.

**Figure 3:**
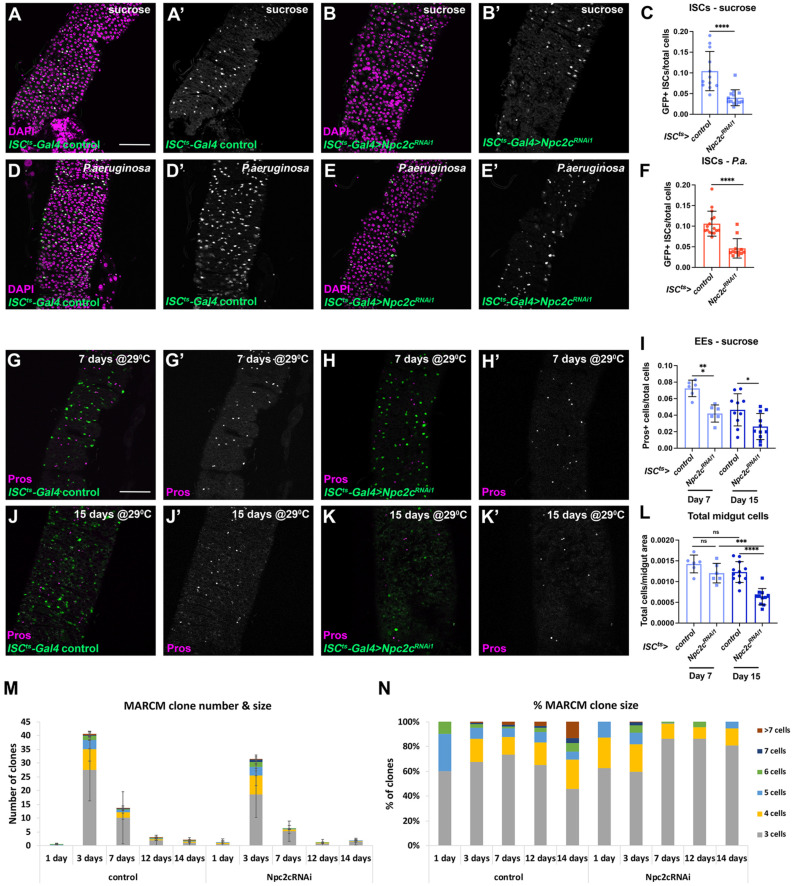
***Npc2c* regulates midgut mitosis and intestinal homeostasis.** (**A**,**B**) Examples of *ISC^ts^*-*Gal4* control and *ISC^ts^*-*Gal4* > *Npc2c^RNAi1^* posterior midguts in uninfected conditions (suc, sucrose feeding), highlighting the GFP-positive ISCs (green in (**A**,**B**) and grey in (**A’**,**B’**)) and stained for the nucleus marker DAPI (magenta). (**C**) Quantification of GFP-positive ISCs from the genotypes shown in (**A**,**B**). (**D**,**E**) Examples of *ISC^ts^*-*Gal4* control and *ISC^ts^*-*Gal4* > *Npc2c^RNAi1^* posterior midguts (**A**,**B**) in *P.a*.-infected conditions, highlighting the GFP-positive ISCs (green in (**D**,**E**) and grey in (**D’**,**E’**)) and stained for the nucleus marker DAPI (magenta). (**F**) Quantification of GFP-positive ISCs from the genotypes shown in (**D**,**E**). (**G**,**H**) Examples of *ISC^ts^*-*Gal4* control and *ISC^ts^*-*Gal4* > *Npc2c^RNAi1^* posterior midguts with transgene induction at 29 °C for 7 days in uninfected conditions, highlighting the GFP-positive ISCs (green in (**G**,**H**) and grey in (**G’**,**H’**)) and stained for the nucleus marker DAPI (magenta). (**I**) Quantification of the ratio of Pros-positive EEs to the total cell number in the conditions shown in (**G**,**H**,**J**,**K**). (**J**,**K**) Examples of *ISC^ts^*-*Gal4* control and *ISC^ts^*-*Gal4* > *Npc2c^RNAi1^* posterior midguts with transgene induction at 29 °C for 15 days in uninfected conditions, highlighting the GFP-positive ISCs (green in (**J**,**K**) and grey in (**J’**,**K’**)) and stained for the nucleus marker DAPI (magenta). (**L**) Quantification of the total number of cells per midgut area for the conditions shown in (**G**,**H**,**J**,**K**). (**M**) Quantification of the number of MARCM clones per midgut and the size of individual lineages for control (genotype *w hsFLP tub*-*Gal4 UAS*-*nlsGFP/+*; *FRT82B tub*-*Gal80/FRT82B arm*-*lacZ*) and *Npc2c^RNAi1^* (genotype *w hsFLP tub*-*Gal4 UAS*-*nlsGFP/+*; *UAS*-*Smvt^RNAi1^/+*; *FRT82B tub*-*Gal80/FRT82B arm*-*lacZ*) MARCM clones 1, 3, 7, 12, and 14 days post heat shock/induction. (**N**) Plotting of the percentage of cell numbers in individual lineages in control and *Npc2c^RNAi1^* MARCM clones 1, 3, 7, 12, and 14 days post heat shock/induction. Experiments were repeated at least twice. Error bars correspond to the standard deviation. Scale bars, 75 μm. Student’s *t*-test was used to test significance in (**C**,**F**,**I**) and one-way ANOVA for multiple comparisons was used in (**L**). ns, not significant; * 0.01 < *p* ≤ 0.05, ** 0.001 < *p* ≤ 0.01, *** 0.0001 < *p* ≤ 0.001, **** *p* ≤ 0.0001.

Increased ISC mitosis is a protective mechanism against infection that promotes survival upon oral *P.a.* administration [51]. To assess whether mitosis is affected in *Npc2c*-silenced midguts, we specifically silenced *Npc2c* in different intestinal populations, in baseline conditions and upon oral *P.a.* infection. First, we used the ISC/EB-specific *esg^ts^*-*Gal4* driver to label intestinal progenitor cells with EGFP and simultaneously silence *Npc2c* in the adult midgut with each of the two RNAi lines. Strikingly, ISC/EB-specific *Npc2c* knockdown inhibited intestinal mitosis almost completely in baseline conditions and upon *P.a.*-induced regeneration (Figure 2A). To further dissect the adult midgut cellular specificity of *Npc2c* function, we silenced *Npc2c* in ISCs using *ISC^ts^*-*Gal4*, EBs using *Su*(*H*)*^ts^*-*Gal4* (*Su*(*H*)-*Gal4 *tub**-Gal80^ts^ *UAS*-EGFP) and ECs using *Myo1A*^ts^-Gal4 (*Myo1A*-Gal4 tub-Gal80^ts^ *UAS*-EGFP). We found that ISC-specific *Npc2c* knockdown caused a dramatic reduction of ISC mitosis phenocopying the *esg*^ts^-Gal4-driven *Npc2c* knockdown (Figure 2B), whereas EB- or EC-specific *Npc2c* silencing had milder effects on ISC mitosis (Figure 2C,D). Since, in flies, the generation rate of differentiated midgut cells is adapted to ISC number changes to maintain homeostasis [64,65], we assessed how regeneration failure due to ISC-specific *Npc2c* deficiency affected different intestinal cell populations and the ISC/differentiated cell balance. When *Npc2c* was silenced specifically in the ISCs, using the *ISC^ts^*-*Gal4*, we observed fewer GFP-positive ISCs compared to the control (Figure 3A,B,D,E). Cell quantifications in control and *Npc2c*-deficient flies underscored the significant reduction of ISCs in both uninfected and *P.a.*-infected posterior midguts (Figure 3C,F). To assess cell differentiation in *Npc2c*-depleted midguts with reduced ISCs, we first quantified Prospero (Pros)-positive cells which correspond to the EE lineage. We found that ISC-specific *Npc2c* silencing for 7 days led to reduced EEs in the posterior midgut of uninfected flies (Figure 3G,J,I). Since the *Drosophila* intestinal epithelium is renewed approximately every two weeks [66], we also assessed EE numbers on day 15 post-*Npc2c^RNAi^* induction and found that EEs were further reduced (Figure 3H,I,K). Importantly, the total number of intestinal cells, stained by the nucleus marker DAPI was not significantly altered upon ISC-specific *Npc2c* silencing on day 7 post-transgene induction. However, on day 15 post-*Npc2c^RNAi^* induction, the total midgut cell number was significantly reduced compared to the control (Figure 3L), indicating that, similar to EEs, the differentiated ECs were also reduced upon *Npc2c* knockdown.

ISCs of *Npc2c*-silenced midguts could be lost due to cell death or because of problematic maintenance. First, to assess the kinetics of ISC mitosis loss, we performed a time-course experiment with and without *p.a.* infection. We found that ISC mitosis was gradually reduced starting from 16 h after initiation of *Npc2c^RNAi^* induction, and by 36 h, it was eliminated (Figure S2A). Thus, we wanted to assess cell maintenance effects before mitosis elimination and focused at 24 h post-*Npc2c^RNAi^* induction. To assess cell death, we stained control and *Npc2c*-silenced midguts with an antibody detecting the cleaved form of Caspase-3. We found no difference in apoptosis in uninfected control and *Npc2c*-silenced midguts (Figure S2B,C) (we occasionally detected Caspase-3-positive cells, as previously reported [66]). Infected control and *Npc2c*-silenced midguts exhibited increased apoptosis, which was restricted to differentiated ECs, but not ISCs (Figure S2D,E) (as previously reported [52]). To assess the role of *Npc2c* in ISC mitosis and maintenance, we generated positively marked *Npc2c*-deficient midgut clones using Mosaic Analysis with a Repressible Cell Marker (MARCM) method [57]. We assessed the number of clones per midgut and the number of cells per clone in control and *Npc2c^RNAi^* lineages at different time points (Figure 3M,N). Interestingly, we found that *Npc2c^RNAi^* and control clones were generated at similar frequencies (Figure 3M). Furthermore, the number of clones increased between day 1 and day 3 by approximately 20-fold and 15-fold in the two genotypes and then was gradually decreased at a similar rate. Strikingly, we observed a growth impairment in the *Npc2c*-deficient clones, contrary to the wild-type ISC clones, which, as expected, were increasing in size at later time points. For example, on day 14 post-induction, no *Npc2c^RNAi^* clone contained more than 5 cells, whereas more than 25% of control clones included 6 cells or above (Figure 3N). Therefore, we conclude that *Npc2c* is necessary for ISC mitosis and maintenance of the stem cell lineage.

**Figure 4:**
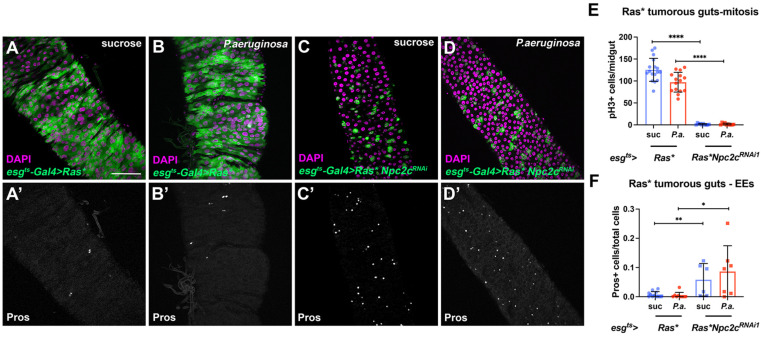
***Npc2c* is necessary for intestinal tumorigenesis.** (**A**–**D**) Examples of posterior *esg^ts^*-*Gal4* > *Ras^Q13^* tumorous midguts without (**A**,**B**) and with *Npc2c^RNAi1^* (**C**,**D**) without (suc, sucrose feeding) (**A**,**C**) and with *P.a.* oral infection (**B**,**D**) stained with DAPI (magenta) and Pros (separate grey channel in (**A’**–**D’**). Scale bars, 75 μm. (**E**) Quantification of mitotic pH3-positive cells in tumorous midguts without and with *Npc2c^RNAi1^* without (blue bars) and with *P.a.* oral infection (red bars). (**F**) Quantification of Pros-positive EEs in tumorous midguts without and with *Npc2c^RNAi1^* without (blue bars) and with *P.a.* oral infection (red bars). Experiments were repeated at least twice. Error bars correspond to the standard deviation. Student’s *t*-test and the Mann–Whitney U-test were used to test significance in (**E**) (N ≥ 12 for each condition) and (**F**) (N ≥ 6 for each condition), respectively. ns, not significant; * 0.01 < *p* ≤ 0.05, ** 0.001 < *p* ≤ 0.01, **** *p* ≤ 0.0001.

Since our results indicate that *Npc2c* regulates ISC mitosis, and we know that stem cell mitosis and cancer are intimately connected [51,67], we assessed the effects of *Npc2c* silencing in Ras-tumorous intestines. By expressing *Npc2c^RNAi^* in tumorous midguts overexpressing through *esg^ts^*-*Gal4* an activated form of *Drosophila* Ras (Ras^Q13^), we found that *Npc2c* silencing decreased tumor mitosis and growth. More specifically, *esg^ts^* > *Ras^Q13^* tumors developed rapidly, populating the whole midgut 3 days post-induction at 29 °C, independent of *P.a.* infection (Figure 4A,B). In contrast, *Npc2c*-silenced *esg^ts^* > *Ras^Q13^* tumors exhibited reduced size (Figure 4C,D) accompanied by dramatically decreased mitosis: ~10-fold reduction in pH3-positive cells with or without the synergistic effect of *P.a.* (Figure 4E). In addition, although *esg^ts^* > *Ras^Q13^* tumors promote EC rather than EE differentiation causing a reduction of EEs [52], we found that tumor-specific *Npc2c* silencing resulted in increased EE numbers by ~8-fold and ~5-fold in the presence and absence of bacterial synergy, respectively (Figure 4F). Overall, these data indicate that *Npc2c* acts in tumor cells to regulate tumorigenesis.

### 3.3. Npc2c Silencing Affects Expression of Mitotic Regulators and Leads to Intestinal Dysbiosis

**Figure 5:**
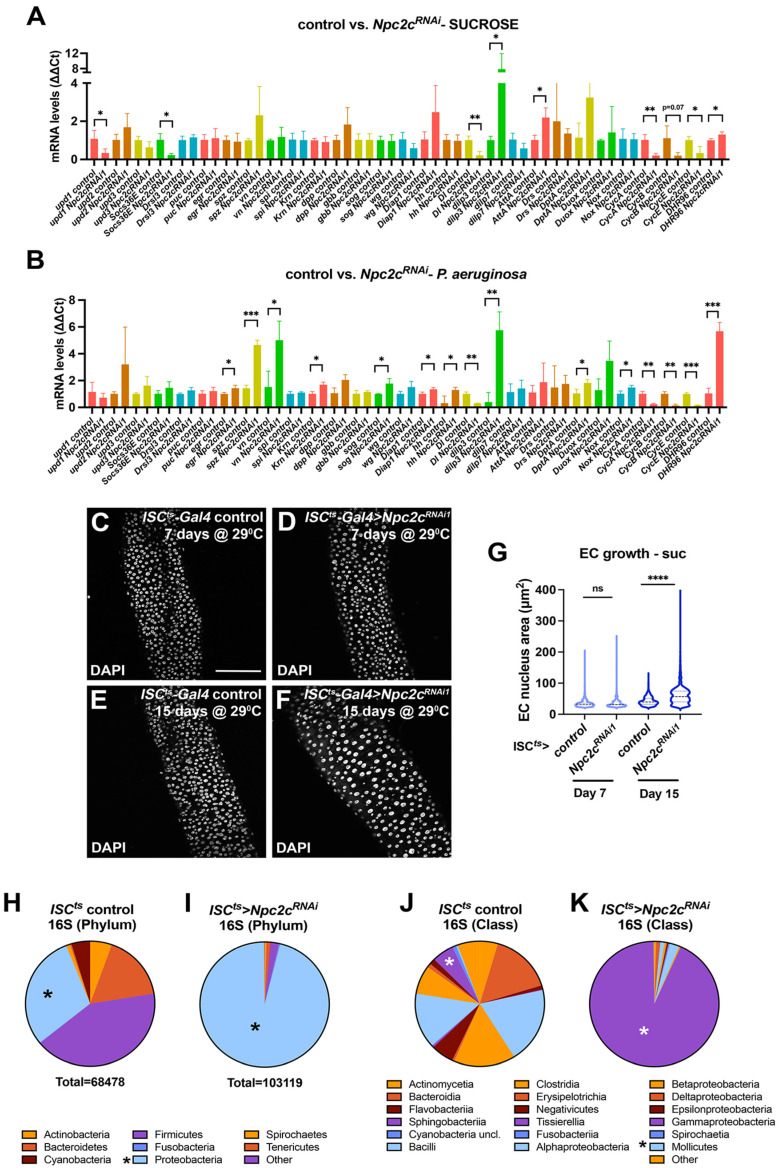
***Npc2c*-silenced midguts exhibit reduced expression of mitosis regulators, increased EC endoreplication and intestinal dysbiosis.** (**A**,**B**) RT-qPCR analysis of candidate genes in *esg^ts^*-*Gal4* control and *Npc2c^RNAi1^* midguts in uninfected (suc, sucrose) (**A**) and *P.a.*-infected conditions (**B**). Bars indicate the mean of at least 3 biological replicates. (**C**–**F**) Examples of *ISC^ts^*-*Gal4* control (**C**,**E**) and *ISC^ts^*-*Gal4* > *Npc2c^RNAi1^* (**D**,**F**) posterior midguts upon transgene induction at 29 °C for 7 days (**C**,**D**) and 15 days (**E**,**F**) in uninfected conditions. The DAPI channel highlighting all midgut nuclei is shown in grey in (**C**–**F**). (**G**) Quantification of EC growth through measurement of the EC nucleus cross-sectional area from maximum intensity projections of DAPI images (as shown in (**C**–**F**)) of *ISC^ts^*-*Gal4* control and *ISC^ts^*-*Gal4* > *Npc2c^RNAi1^* at 7 and 15 days of induction at 29 °C. (**H**,**I**) Bacterial composition (phyla) from dissected midguts determined by 16S rDNA sequencing in uninfected *esg^ts^*-*Gal4* control (**H**) and *Npc2c^RNAi1^* (**I**) midguts. (**J**,**K**) Bacterial composition (Class) from dissected midguts determined by 16S rDNA sequencing in uninfected *esg^ts^*-*Gal4* control (**J**) and *Npc2c^RNAi1^* (**I**) midguts. Sequencing in (**H**–**K**) was performed in biological replicates and the data were merged. Scale bars, 75 μm. Error bars correspond to the standard deviation. Statistical significance was tested with Student’s *t*-test or the Mann–Whitney U-test in (**A**,**B**) (N = 3 or N = 6) and Student’s *t*-test for G (N = 50). ns, not significant; * 0.01 < *p* ≤ 0.05, ** 0.001 < *p* ≤ 0.01, *** 0.0001 < *p* ≤ 0.001, **** *p* ≤ 0.0001.

To assess whether conserved signaling pathways and genes with known roles in the *Drosophila* midgut might be implicated in the *Npc2c*-silencing phenotypes, we performed gene expression analysis using RT-qPCR in control and progenitor-specific *Npc2c*-silenced uninfected and *P.a.*-infected midguts. Specifically, we assessed expression of genes implicated in ISC proliferation and differentiation, including members of the Jak/Stat, EGFR, JNK, Notch, Wg and Hippo pathways, immunity pathways, as well as mitosis regulators. We also tested expression of the *DHR96* gene, which is involved in sterol homeostasis (Figure 5A,B). In agreement with the severe mitosis impairment caused by *Npc2c* knockdown, we observed significantly lower expression of mitotic cyclins in *Npc2c*-silenced midguts in both uninfected and *P.a*.-infected conditions. Specifically, the mRNA expression levels of three cyclins, *CycA*, *CycB* and *CycE,* were reduced by more than 5-fold. In addition, expression of the Notch ligand *Delta* (*Dl*), which marks the ISCs and mediates their differentiation [68], was dramatically reduced in *Npc2c*-silenced midguts. Altogether, the reduced expression of *Cyclin* genes and *Dl* in *Npc2c*-silenced midguts compared to wild-type controls is indicative of the impaired ISC mitosis and the decreased ISC numbers accompanying *Npc2c* knockdown.

Interestingly, in uninfected, but not *P.a.*-infected midguts, the mRNA level of the Jak/Stat ligand Unpaired 1 (Upd1) and the direct target of the pathway, Socs36E, were downregulated in progenitor-specific *Npc2c*-silenced midguts (Figure 5A,B). Since the Upd1-Jak/Stat pathway controls basal turnover of the midgut epithelium [69], we hypothesized that *Npc2c* may regulate Upd1 activity and therefore ISC proliferation in baseline, uninfected conditions. In *Npc2c*-deficient flies, other genes such as the *Drosophila* insulin-like peptide 3 (*Dilp3*) and the antimicrobial peptide Attacin A (*AttA*) were significantly upregulated in uninfected conditions (Figure 5A), whereas in *P.a.*-infected midguts, several genes encoding ligands that control ISC proliferation/differentiation and host defense were upregulated. These included genes encoding the EGFR pathway ligands *vein* (*vn*) and Keren (*Krn*), the *Drosophila* TNF-α ligand *eiger* (*egr*), the Insulin receptor ligand *Dilp3*, Hedgehog (*hh*), and the Hippo pathway effector *Drosophila* inhibitor of apoptosis 1 (*Diap1*). In addition, genes encoding the IMD/antimicrobial peptide Diptericin A (*DptA*), the NADPH oxidase *Nox*, and the Toll pathway ligand, Spatzle (*spz*) were also found increased in *P.a.*-infected *Npc2c*-silenced midguts (Figure 5B). Finally, expression of *DHR96* was induced upon *Npc2c* silencing in both uninfected and *P.a.*-infected conditions, indicating a feedback regulation between Npc2c and DHR96 (Figure 5A,B).

ISC elimination and decrease in ISC mitosis via *CycE* downregulation can trigger compensatory EC polyploidization [51]. Since progenitor-specific *Npc2c* silencing blocked mitosis almost completely and reduced *CycE* expression significantly, we tested EC ploidy in control and ISC-specific *Npc2c*-silenced midguts. To assess EC ploidy, we measured the EC nucleus maximum cross-sectional area, which was previously shown to strongly correlate with EC DNA content [51]. Specifically, we imaged intestinal nuclei and measured EC nucleus size in adult wild-type and ISC-specific *Npc2c*-silenced midguts in baseline conditions on day 7 and day 15 upon *UAS*-*Npc2c^RNAi^* induction. We observed that *Npc2c*-silenced midguts did not exhibit a significant difference in the EC nucleus area on day 7 compared to control midguts (Figure 5C,D,G). However, ECs were significantly enlarged on day 15 (Figure 5E–G). Nevertheless, this compensatory EC endoreplication upon *Npc2c* silencing did not suffice for tissue maintenance and survival upon infection (Figure 1H).

Previous in vitro experiments in cultured S2 cells showed that over-expression of several *Npc2* genes activated IMD and stimulated *DptA* expression [48]. Interestingly, we showed that ISC/EB-specific *Npc2c* knockdown upregulated expression of *AttA* (Figure 5A), an AMP known to be induced in the midgut in response to Gram-negative bacteria [70]. Thus, we hypothesized that ISC/EB-specific *Npc2c* silencing may induce dysbiosis in the midgut, which activates the IMD pathway and AMP transcription. To address this hypothesis, we performed 16S rDNA sequencing of uninfected control and uninfected ISC/EB-specific *Npc2c*-silenced midguts in biological replicates. We found that *Npc2c*-silenced midguts encompassed increased numbers of commensal bacteria (103119 compared to 68478 identified in the control midguts). Furthermore, the microbiome of *Npc2c*-silenced midguts exhibited decreased complexity. For example, common phyla present in control flies, such as Actinobacteria, Bacteroidetes and Firmicutes, were significantly decreased in *Npc2c*-silenced flies, whereas, the abundance of other commensals, such as Proteobacteria, was increased from 30% to 95% (Figure 5H,I). Specifically, *Npc2c*-deficient midguts favored the growth of Gram-negative gamma-proteobacteria (Figure 5J,K), and specifically the species *Gilliamena intestini*. To validate the metagenomics results and isolate candidate species involved in dysbiosis, we assessed changes in the abundance of commensal bacteria in independent experiments by counting colony-forming units (CFUs) of wild-type and *Npc2c*-silenced flies in uninfected conditions. Using a nutrient-rich medium, brain heart infusion (BHI), that allows growth of various hard-to-grow microorganisms, we confirmed an increase in the abundance of commensals upon *Npc2c* knockdown, albeit not statistically significant, as well as reduced complexity in the types of isolated colonies in anaerobic conditions (not shown). In particular, colonies with five and three distinct morphological characteristics, were isolated from control and *Npc2c*-silenced midguts, respectively. Colony PCR followed by 16S rDNA gene sequencing identified common commensals, including *Lactobacillus* spp. (*L. plantarum* and *L. brevis*) and *Enterococcus* spp. (*Enterococcus termitis*) in control and *Npc2c*-silenced flies, but proteobacteria could not be isolated. Overall, these findings highlight the significance of *Npc2c* in microbiota homeostasis, which is responsible for the induction of host defense mechanisms and epithelial homeostasis.

### 3.4. ISC-Specific Npc2c Silencing Impairs Cholesterol Trafficking and Controls Ecdysone Signaling

**Figure 6:**
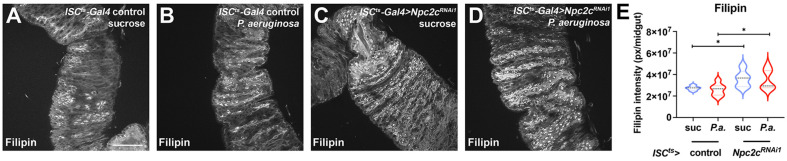
***Npc2c*-silenced midguts exhibit increased sterol aggregation.** (**A**–**D**) Examples of *ISC^ts^*-*Gal4* control (**A**,**B**) and *ISC^ts^*-*Gal4* > *Npc2c^RNAi1^* (**C**,**D**) posterior midguts in uninfected (sucrose feeding) (**A**,**C**) and *P.a.*-infected (**B**,**D**) conditions stained with filipin (in grey) to highlight sterol aggregates. (**E**) Quantification of the filipin fluorescent signal from images like those shown in (**A**–**D**). Experiments were repeated at least twice. Scale bars, 75 μm. Error bars correspond to the standard deviation. Statistical significance in (**E**) was tested with Student’s *t*-test (N ≥ 10). ns, not significant; * 0.01 < *p* ≤ 0.05.

Previous gene expression studies support a specific role of the posterior midgut in lipogenesis and the processing of specific lipids, including sphingolipids and cholesterol [13,61,71]. Importantly, *Npc2c* together with the gene encoding the cholesterol acyltransferase (ACAT) were found to be selectively expressed in the posterior midgut [13]. To assess whether *Npc2c* controls cholesterol accumulation, we stained control and *Npc2c*-deficient midguts with Filipin. Filipin labels non-esterified sterols and is commonly used to study sterol accumulation in mammalian cells, but has also been used to assess *Npc1a* mutants in *Drosophila* [21,72]. We found aberrant accumulation of free cholesterol in both uninfected (Figure 6A,C) and *P.a.*-infected midguts (Figure 6B,D) upon ISC-specific *Npc2c* knockdown (Figure 6A–E). This result underscores a critical role of Npc2c in intestinal sterol distribution.

**Figure 7:**
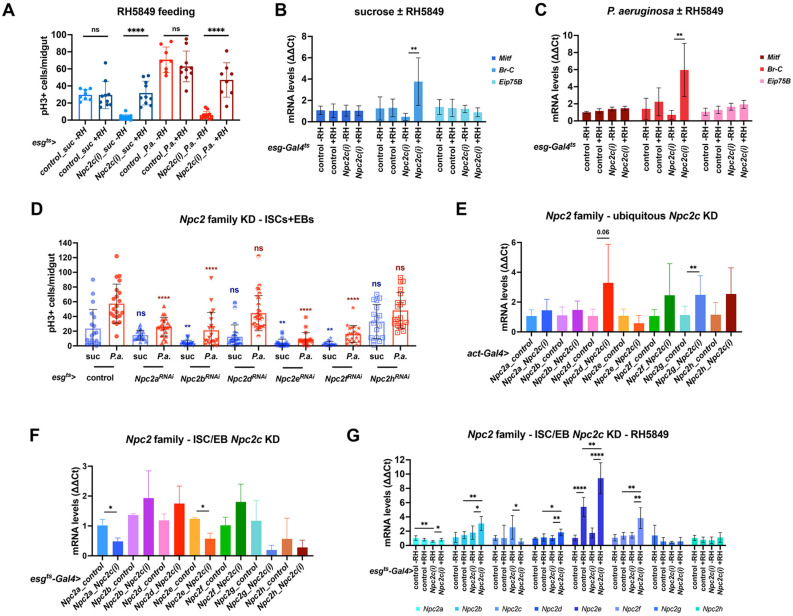
***Npc2c* mediates 20E signaling and interacts with other *Npc2* family members.** (**A**) Quantification of the mitotic index (pH3-positive cells) in *esg^ts^*-*Gal4* control and *esg^ts^*-*Gal4* > *Npc2c^RNAi1^* uninfected (suc, sucrose feeding) and *P.a.*-infected adult midguts upon supplementation of the 20E agonist RH5849. (**B**,**C**) RT-qPCR analysis of *Mitf*, *Br* and *Eip75B* in *esg^ts^*-*Gal4* control and *esg^ts^*-*Gal4* > *Npc2c^RNAi1^* midguts in the presence of absence of RH5849 in uninfected (**B**) and *P.a.*-infected conditions (**C**). (**D**) Quantification of the mitotic index in adult midguts of *esg^ts^*-*Gal4* control and *Npc2^RNAi^* family members (*Npc2a*, -*b*, -*d*, -*e*, -*f*, -*h*) in uninfected (blue bars) and *P.a.*-infected (red bars) conditions. (**E**) RT-qPCR analysis of *Npc2* gene family (*Npc2a*-*h*) in *Act5C*-*Gal4* control and *Act5C*-*Gal4* > *Npc2c^RNAi1^* third instar larvae. (**F**) RT-qPCR analysis of *Npc2* gene family (*Npc2a*-*h*) in *esg^ts^*-*Gal4* control and *esg^ts^*-*Gal4* > *Npc2c^RNAi1^* midguts in uninfected conditions. (**G**) RT-qPCR analysis of *Npc2* gene family (*Npc2a*-*h*) in *esg^ts^*-*Gal4* control and *esg^ts^*-*Gal4* > *Npc2c^RNAi1^* midguts in the presence or absence of RH5849 in uninfected conditions. Experiments were repeated at least twice. Error bars correspond to the standard deviation. Statistical significance was tested with the Mann–Whitney U-test in (**A**) (N ≥ 8), (**B**,**C**,**E**–**G**) (N = 6) and Student’s *t*-test in (**D**) (N ≥ 12). ns, not significant; * 0.01 < *p* ≤ 0.05, ** 0.001 < *p* ≤ 0.01, **** *p* ≤ 0.0001.

Sterol transport to the ER/mitochondria controls synthesis of 20E, larval molting and metamorphosis [73]. In addition, ovary-produced 20E activates the Ecdysone receptor (EcR) signaling cascade in the neighboring posterior midgut ISCs, which, in turn, promotes Ecdysone-induced-protein-75B (Eip75B)-mediated EB to EC differentiation [31]. Here, we tried to rescue the mitosis impairment of *Npc2*-deficient progenitors by supplementing the fly diet either with 250 ug/mL cholesterol or with 0.008 mg/g 20E. We found that cholesterol did not rescue mitosis (Figure S3A), probably because *Npc2c* deficiency impaired its trafficking. Also, we found that 20E administration could not rescue mitosis (Figure S3B). Since 20E is easily metabolized and cleared by the gut, we switched to the non-steroidal EcR agonist RH5849, which is highly stable, specific and has higher efficacy compared to 20E [31,74,75]. Indeed, supplementation of the fly diet with RH5849 at 50 ug/mL fully rescued the mitosis impairment caused by *Npc2c* silencing, reflected by ~10- and ~9-fold increase in the mitotic index of *Npc2c*-deficient flies in uninfected and *P.a.*-infected midguts, respectively (Figure 7A). Binding of 20E to EcR triggers the activation of a small set of primary response genes, many of which encode transcription factors [76]. To understand how *Npc2c* impinges on 20E signaling to control ISC activity, we assessed the expression of two known EcR targets: the transcription factor *Broad* (*Br*) and the nuclear receptor and *PPARγ*-homologue, *Eip75B*, upon progenitor-specific *Npc2c* silencing in the presence or absence of infection, with or without RH5849 supplementation. We also assessed expression of the *Drosophila* ortholog *PPARγ*-dependent Transcription Factor EB (TFEB), *Mitf*, which, like TFEB [77], is involved in the lysosomal–autophagy pathway [78]. In both uninfected and *P.a*.-infected *Npc2c*-silenced midguts, *Br*, but not *Eip75B* or *Mitf,* was significantly reduced, and its expression was significantly increased upon supplementation with RH5849 (Figure 7B,C). However, in uninfected flies, the expression levels of *Eip75B* and *Mitf* remained unchanged upon *Npc2c* silencing and the supplementation of RH5849 (Figure 7B); *P.a.* infection showed tentatively lower expression in untreated *Npc2c*-silenced midguts, which was increased upon RH5849 supplementation (Figure 7C). Overall, these results highlight that Npc2c-transported sterol controls the EcR pathway, which, through its effector Br, regulates ISC mitosis by acting downstream or in parallel to Npc2c.

### 3.5. Additional Npc2 Family Genes Control Intestinal Mitosis

Interestingly, another member of the Npc2 family, *Npc2b*, was previously shown to transport sterol in the midgut and its ISC-specific silencing significantly reduced EEs [13], a phenotype reminiscent of *Npc2c* silencing. To examine whether *Npc2b* and other *Npc2* family members act similarly to *Npc2c* in intestinal progenitors, we specifically silenced *Npc2a*, -*b*, -*d*, -*e*, -*f*, -*h* (an RNAi line for *Npc2g* was not available) in adult midgut progenitors using *esg^ts^*-*Gal4* and we quantified mitosis in uninfected and *P.a*.-infected conditions. We found that *Npc2b*, -*e*, -*f* knockdown significantly decreased mitosis in uninfected and *P.a*.-infected midguts, whereas *Npc2a* silencing only mildly reduced mitosis in *P.a.*-infected midguts (Figure 7D). On the contrary, *Npc2d* and *Npc2h* silenced midguts exhibited no difference in mitosis (Figure 7D). Since *Npc2* family genes exhibit broad and specific expression patterns, but their expression might be cross-regulated, we decided to assess the expression of *Npc2* genes in animals with ubiquitous downregulation of *Npc2c*. We found that ubiquitous *Npc2c* silencing in larvae induced the expression of *Npc2d*, -*f*, -*g* and -*h* (Figure 7E). This phenotype indicates strong redundancy between *Npc2c* and other *Npc2* family members. To assess whether this is also true in the adult intestine, where the expression of *Npc2* genes varies in different cell types and upon infection challenge (Figure S1), we performed RT-qPCR of control and ISC/EB-specific *Npc2c*-silenced adult midguts in uninfected conditions. We found that the levels of *Npc2a* and *Npc2e* mRNA were significantly downregulated upon progenitor-specific *Npc2c* silencing (Figure 7F).

To further characterize how *Npc2* family members correlate to *Npc2c* and the 20E pathway, we assessed their expression upon RH5849 supplementation in the absence of bacterial infection. In the absence of the chemical, we observed similar results as those described previously (Figure 7F), with the exception of the *Npc2e* expression, which behaved opposite upon *Npc2c*-silencing. In uninfected conditions, administration of RH5849 strikingly induced *Npc2e* expression in control flies, whereas RH5849 significantly increased the expression of *Npc2b*, *Npc2e* and *Npc2f* in *Npc2c*-silenced flies compared to mock control. *Npc2f* appeared to be significantly overexpressed in both mock and drug-administered flies (Figure 7E). Conclusively, these results indicate that not only *Npc2c* expression, but also other *Npc2* genes, which might act synergistically, are also regulated by the ecdysone pathway.

## 4. Discussion

NPCs are intracellular sterol-binding and sterol-transporting proteins, with Npc1 encompassing a transmembrane domain unlike Npc2 [79]. Specifically, human Npc2 localizes in puncta that co-stain with the lysosomal marker cathepsin D and the late lysosomal/endosomal marker Lamp-1 in cultured fibroblasts [80]. We found that *Drosophila* Npc2c staining is also punctate, but unlike its human counterpart, it does not colocalize with the Lamp-1 marker in midgut progenitors (Figure 1A). Intriguingly, *Npc2e*, the closest fly paralogue to *Npc2c*, encodes a secreted protein with the ability to bind bacterial components and activate AMPs upon overexpression in S2 cells [48]. This raises the question whether the midgut Npc2c puncta correspond to extracellular accumulations. Future experiments will resolve the precise cellular localization of Npc2c.

To investigate whether silencing of *Npc2c* might affect expression of the other seven *Npc2* family members, we ubiquitously downregulated *Npc2c* in larvae and found that most *Npc2* mRNAs (except *Npc2e*) tend to increase, with *Npc2g* increasing most significantly (Figure 7E). The *Npc2c*-silenced larvae develop into adults, indicating that *Npc2c* is not necessary for development and this may be due to possible redundancy between *Npc2c* and other *Npc2′s*. Interestingly, ISC/EB-specific *Npc2c* knockdown does not broadly induce *Npc2* gene expression. In fact, we observe significant reduction of *Npc2a* and *Npc2e* in the adult midgut. Given that ISC/EB-specific silencing of several *Npc2* genes reduces ISC mitosis, the observed effects on mRNA expression indicate potential cross regulation. It is worth noting that ISC/EB-*Npc2e* silencing phenocopies *Npc2c* loss and eliminates midgut mitosis in both uninfected and *P.a.*-infected conditions (Figure 7D). Our results also underscore the role of *Npc2c* in midgut sterol availability and subsequent ecdysone action. We found that administration of the ecdysone agonist RH5849 rescues the mitosis impairment caused by the ISC/EB-specific *Npc2c* silencing, potentially through expression of the Br transcription factor (Figure 7A–C). Interestingly, we also found that RH5849 directly induces *Npc2e* in wild-type midguts, and this is enhanced in ISC/EB *Npc2c*-silenced midguts. Simultaneously, *Npc2a* appears to be downregulated in *Npc2c*-silenced flies treated with vehicle, and its expression is rescued by RH5849 administration (Figure 7E). Given that *Npc2a* is also necessary for midgut mitosis upon *P.a.* infection, it seems that 20E signaling mediates *Npc2c* action in the midgut. Nevertheless, the interplay of fly *Npc2* genes remains unclear. It is likely that different cholesterol substrates may be able to bind Npc2 proteins, contributing to homeostatic cholesterol metabolism. For example, the human Npc2 protein exists in different glycoforms that bind different sterols, such as cholesterol, oxysterols, and plant sterols [81]. However, it is not yet known whether the fly Npc2 protein repertoire is so broad to mimic the different glycoforms observed in humans, or to bind specific kinds of sterols more than others.

Since we silenced *Npc2c* in intestinal progenitors, we would not expect an effect on the production of 20E, which occurs in the ovaries of mated flies [31]. This is evident by the unchanged expression of *Eip75B*, which is a canonical early 20E target gene [82], in control and experimental flies. It is therefore a mystery why *Br* is downregulated upon *Npc2c* silencing in the midgut progenitors while it is upregulated via the administration of the 20E agonist RH5849. A possible explanation could be that without the drug, progenitor numbers decrease in *Npc2c*-silenced flies (Figure 3C,F). According to FlyGutSeq [61], *Br* is expressed more in ISCs and less in EBs at basal conditions, while its expression in EBs goes up by 27.5-fold upon *P. entomophila* infection. The loss of progenitors in our experimental flies could therefore explain the reduction of *Br*. The rescue of mitosis, and as such the integrity of ISCs, may explain the restoration of *Br* levels. However, we cannot rule out possible modifications in its expression by cholesterol trafficking. More research is needed to decipher the role of cholesterol intracellular transport in modifying the expression of *Br* and elucidate the relationship between *Br* and the fly *Npc2* genes. This could help us understand the axis of steroid availability, cholesterol metabolism, and cellular membrane integrity, and its roles in cellular division.

Understanding the role of cholesterol in cellular division is important not only in degenerative diseases such as Neimann–Pick type-C disease, but also in the context of cancer. Cholesterol has been shown to contribute to cancer growth through the phosphoinositide-3 kinase (PI3K)/protein kinase B (AKT) pathway [83]. Lovastatin, an inhibitor of the rate-limiting cholesterol synthesis enzyme 3-hydroxy-3-methyl-glutaryl-CoA (HMG-CoA) reductase, downregulates PI3K, AKT, and mammalian target of rapamycin (mTOR) in colorectal cancer cells via a phosphatase and tensin homologue (PTEN)- and bone morphogenetic protein (BMP)-dependent manner [84]. A small pilot study shows that Simvastatin administration recapitulates the downregulation of phosphorylated mTOR and induces a tentative increase in BMP in colorectal cancer patients [84]. Furthermore, an analysis of 999 colorectal cancer patients shows reduced death incidence for patients taking statins, which benefits more the patients who have tumors with intact BMP signaling [85]. Although a meta-analysis shows statins to be effective in preventing and treating colorectal cancer [86], patients with previously treated metastatic disease do not appear to benefit from a combinatorial regimen of chemotherapy and statins [87]. Here, we show that targeting cholesterol trafficking is an effective way of blocking mitosis in transformed Ras^V12^ cells (Figure 4) as well as healthy progenitors in the fly midgut (Figure 2A,B), revealing an attractive avenue of future research for intestinal cancer treatment. Despite previous research showing that *Npc* genes are not associated with Alzheimer’s disease (AD) [88], efforts to target cholesterol trafficking as anti-cancer therapy will have to consider possible wide-spread effects not only in ISCs, but also in the brain, since cholesterol metabolism deregulation and cholesterol penetration through a disrupted blood–brain barrier has been suggested to contribute to AD [89,90], a mechanism which could hypothetically be implicated in Parkinson’s disease as well [90,91]. In addition, any interventions involving the *Npc* genes will also have to consider the neurodegenerative effects observed in Niemann–Pick Type C disease [38].

Finally, it is becoming clear that impairment of cholesterol trafficking in the intestine can lead to microbiome alterations. For example, intestinal dysbiosis has been shown in *Npc1* mutant Balb/c mice, exhibiting increased proteobacteria and cyanobacteria [92]. Our results agree with the role of *Npc* genes in maintaining a normal gut microbiome, as we also observed an increase in proteobacteria upon *Npc2c* silencing (Figure 5H–K). Interestingly, proteobacteria have been associated with human disease [93]. The upregulation of the AMP AttA (Figure 4) in *Npc2c*-silenced midguts indicates the presence of a pathogenic organism and lets us speculate that the increase in gamma-proteobacteria may be a sign of pathogenicity. Since it has been shown that *Npc2a* and *Npc2e* activate the AMP DptA when stimulated with PGs [48], it would be interesting to investigate if fly *Npc2* genes are also responsible for the upregulation of *AttA*. Additional work is also needed to establish whether the observed microbiome changes are due to loss of progenitor mitosis, aggregation of sterols, or a combination of the two events. It is also unknown whether the disruption of cholesterol trafficking in the *Drosophila* midgut progenitors and sterol aggregation, or the loss of progenitor cells directly give a competitive advantage to proteobacteria or whether the observed impairments result in the death of the lost microbiota.

In conclusion, we found that *Npc2c* loss in ISCs/EBs results in reduced ISC mitosis, impaired cholesterol metabolism, and dysbiosis in *Drosophila*. Mitosis can be rescued by administration of the non-steroidal ecdysone agonist RH5849, which results in overexpression of certain *Drosophila Npc2* family genes. Our results highlight the importance of *Npc2c* for ISC-mediated intestinal homeostasis, and more specifically the necessity of intact cholesterol metabolism in ISCs for effective tissue regeneration. Furthermore, our results indicate redundant roles between *Drosophila Npc2* genes.

## Data Availability

The authors declare that the data supporting the findings of this study are available within the paper and its supplementary information files.

## Supplementary Materials

The following supporting information can be downloaded at https://www.mdpi.com/article/10.3390/metabo13101084/s1. Table S1: Key Resources Table; Table S2: List of primer sequences; Table S3: qPCR amplification program; Table S4: 16S colony PCR amplification program; Figure S1: *Npc2* gene family expression in adult midguts; Figure S2: Progenitor-specific *Npc2c* silencing reduces mitosis effectively, but it does not cause apoptosis; Figure S3: Supplementation of the diet with cholesterol or 20E does not rescue reduced mitosis caused by *Npc2c* silencing.
