# Supplementary material for: The Sterol Transporter Npc2c Controls Intestinal Stem Cell Mitosis and Host–Microbiome Interactions in Drosophila
Source: Metabolites. 2023 Oct 16;13(10):1084. doi: 10.3390/metabo13101084 (PMC10609107; doi:10.3390/metabo13101084)
Supplement: Supplementary file 1 [file metabolites-13-01084-s001.zip › metabolites-2646787-supplementary.pdf]

The sterol transporter Npc2c controls intestinal stem cell mitosis and host-microbiome interactions in *Drosophila*

SUPPLEMENTARY TABLES

Table S1: Key Resources Table

Table S2: List of primer sequences

Table S3: qPCR amplification program

Table S4: 16S colony PCR amplification program

SUPPLEMENTARY FIGURES

Figure S1. *Npc2* gene family expression in adult midguts.

Figure S2. Progenitor-specific *Npc2c* silencing reduces mitosis effectively, but it does not cause apoptosis.

Figure S3. Supplementation of the diet with cholesterol or 20E does not rescue reduced mitosis caused by *Npc2c* silencing.

Table S1: Key Resources Table

REAGENT or RESOURCE	SOURCE	IDENTIFIER
**Antibodies**		
Rabbit anti-Npc2c	Boster	DZ41252
Rabbit anti-phospho Histone H3(Ser10)	Millipore	06-570
Chicken anti-GFP	Invitrogen	A10262
Rabbit anti-GFP	Invitrogen	A6455
Rabbit anti-Cleaved Caspase-3	Cell Signaling	9661
Mouse anti-Prospero	DSHB	MR1A
Mouse anti-Delta	DSHB	C594.9B
Alexa Fluor ™ 488 donkey anti-rabbit IgG	Invitrogen	A21206
Alexa Fluor ™ 488 goat anti-chicken IgG	Invitrogen	A32931
Alexa Fluor™ 555 goat anti-rat IgG	Invitrogen	A-21434
Alexa Fluor™ 555 donkey anti-mouse IgG	Invitrogen	A31570
Alexa Fluor™ 555 donkey anti-rabbit IgG	Invitrogen	A31572
Alexa Fluor™ 555 goat anti-rat IgG	Invitrogen	A-21434
Streptavidin, Cy3	Vector Laboratories	SA-1300
**Bacterial Strains**		
Pseudomonas aeruginosa, PA14	Apidianakis & Rahme, 2009	NA
**Chemicals, Peptides, and Recombinant Proteins**		
Drosophila Agar Type II	Apex	66-103
Cornmeal	Local market	NA
Inactivated dried yeast	Local market	NA
Sucrose	Local market	NA
Tegosept	Apex	20-258
Propionic acid	Scharlau	AC18941000
Acetic acid glacial, ACS reagent, ≥99.7%	Sigma	695092
Luria Broth	Condalab	1231.00
Luria Broth agar	Invitrogen	22700-025
Brain Heart Infusion (BHI) broth	Millipore	110493
Hemin	Sigma	51280
Vitamin K_1	Sigma	V3501
QIAzolÒ Lysis Reagent	QIAGEN	79306
Bovine Serum Albumin	Sigma	A7888
Triton™ X-100	Sigma	T8787
Filipin III from Streptomyces filipinensis	Sigma	F4767
RH5849	DrEhrenstorfer	DRE-C16813000
Cholesterol	Sigma	C8667-500MG
20 Hydroxyecdysone	Sigma	H5142
DAPI	Sigma	D9542-10mg
Vectashield Antifade Mounting Medium	Vector	H-1000
Bromophenol blue	Sigma	114391
KAPA SYBR Fast Master Mix (2x) Universal	KAPA Biosystems	KK4602
UltraPure Distilled Water DNase/RNase Free	Invitrogen	10977-035
Ethanol absolute	Scharlau	ET00072500
Methanol	Honeywell	34860
2-Propanol	Honeywell	24137
Chloroform	Millipore	1.02445.2500
DMSO	Sigma	D2650
Agarose	Cleaver Scientific	9012-36-6
Ethidium bromide solution	Sigma	E-1385
**Critical Commercial Assays**		
RQ1 reagent kit	Promega	M6101
PrimeScript RT Master Mix	Takara	RR036A

KAPA HiFi HotStart Ready Mix PCR Kit	Roche	07958889001
NucleoSpin Gel and PCR Clean up	Macherey-Nagel	740609.50
**Experimental Models: *Drosophila melanogaster* Strains**		
w^{1118}	BDSC	6326
w; esg-Gal4 UAS-GFP tub-Gal80^{ts} (*esg*^{ts}*-Gal4*)	Micchelli & Perrimon, 2006	NA
w; esg-Gal4 UAS-GFP tub-Gal80^{ts}*; Su(H)-Gal80* (*ISC*^{ts}*-Gal4*)	Zheng & Hou, 2015	NA
w; Su(H)-Gal4 UAS-CD8GFP tub-Gal80^{ts20}*/CyO* (*SuH*^{ts}*-Gal4*)	Tamamouna et al, 2020	NA
w tub-Gal80^{ts}*/FM7; Myo1A-Gal4 UAS-EGFP/CyO* (*Myo1A*^{ts}*-Gal4*)	Apidianakis et al, 2009	NA
w hsFLP tub-Gal4 UAS-nls-GFP/FM7; FRT82B tub-Gal80/TM6B	Pitsouli & Delidakis, 2005	NA
w; FRT82B arm-lacZ/TM6B	BDSC	BDSC-7369
w; UAS-Npc2c^{RNAi1}*/CyO; FRT82B arm-lacZ/TM6C*	This study	NA
w; esg-Gal4 UAS-eGFP tub-Gal80^{ts}*/CyO; UAS-Ras1*^{Q13}*/TM6C*	Neophytou & Pitsouli, 2021	NA
w; UAS-Npc2a^{RNAi} (KK108573)	VDRC	v106771
w; UAS-Npc2b^{RNAi} (KK103974)	VDRC	v101233
w; UAS-Npc2c^{RNAi1} (KK103904)	VDRC	v101583
w; UAS-Npc2c^{RNAi2} (GD6798)	VDRC	v31139
w; UAS-Npc2d^{RNAi} (GD5679)	VDRC	v31095
w; UAS-Npc2e^{RNAi} (KK104354)	VDRC	v100445
w; UAS-Npc2f^{RNAi} *(KK111008)*	VDRC	v102172
**Oligonucleotides**		
See Table S2 for primer sequences	Integrated DNA Technologies (IDT)	NA
**Deposited Data**		
Metagenomic sequencing data	https://www.ncbi.nlm.nih.gov/sra/PRJNA804129	PRJNA804129
**Software and Algorithms**		
ImageJ	NIH	https://imagej.nih.gov/ij/
Prism 9	GraphPad	https://www.graphpad.com/scientific-software/prism/
Photoshop 2023	Adobe	https://www.adobe.com/cy_en/products/photoshop.html
BLAST	NCBI	https://blast.ncbi.nlm.nih.gov/Blast.cgi
Microsoft Office 2023	Microsoft	https://www.office.com/
Mann-Whitney U Test Calculator	Social Science Statistics	www.socscistatistics.com
Log-Rank Test	MedCalc statistical software	www.medcalc.org
FlyAtlas2	Leader et al, 2017	https://motif.mvls.gla.ac.uk/FlyAtlas2

Table S2: List of primer sequences

**Primers used for 16S rRNA amplification**			
**Name**		**Sequence**	
27F		AGAGTTTGATCCTGGCTCAG	
1492R		GGTTACCTTGTTACGACTT	
**Primers used in RT-qPCR experiments**			
**Gene Name**	**Gene symbol**	**Forward Primer**	**Reverse Primer**
Alpha tubulin	α-tub	GCTGTTCCACCCCGAGCAGCTGATC	GGCGAACTCCAGCTTGGACTTCTTGC
Attacin A	*AttA*	CACAATGTGGTGGGTCAGG	GGCACCATGACCAGCATT
Broad	*Br*	TAACCTCGGCGTTCGAGAATC	TTTGCAGGGTGTGCTCTTGA
Cyclin A	*CycA*	TCTGGAATATTTCCGGGAGAG	AGCGCATATTGTGGCTAATGT
Cyclin B	*CycB*	GATGCGGCACAGAAAGACTC	TTCTTCCAGTGGCTGTCCA
Cyclin E	*CycE*	ACAAATTTGGCCTGGGACTA	GGCCATAAGCACTTCGTCA
Death-associated inhibitor of apoptosis 1	*Diap1*	CCCCAGTATCCCGAATACGC	TCTGTTTCAGGTTCCTCGGC
decapentaplegic	*dpp*	GTATCTGTGTGCGCTGCTG	CCTAAGCATACTCCTCTTTTCCTC
Delta	*Dl*	GCGACAAGCCCAATCAAT	GGTTCCAGAACGCACTCG
Diptericin A	*Dipt*	GGCTTATCCGATGCCCGACG	TCTGTAGGTGTAGGTGCTTCCC
Drosomycin	*Drs*	GACTTGTTCGCCCTCTTCG	CTTGCACACAGACGACAG
Dual oxidase	*Duox*	CACGCGCAGCAGGATGTAAGGTTT	GCTGCACGCCAACCACAAGAGACT
Ecdysone-induced protein 75B	*Eip75B*	ATGCAACAGAGCACCCAGAAT	CATGGAGTAGGAGGGGCAATC
eiger	*egr*	AGCTGATCCCCCTGGTTTTG	GCCAGATCGTTAGTGCGAGA
glass bottom boat	*gbb*	GAGTGGCTGGTCAAGTCGAA	GAAGCCGATCATGAAGGGCT
hedgehog	*hh*	CAAGGAGCAGTTGCACAGTT	TGTAGAGCGCATTGGCATAC
Insulin-like peptide 3	*Dilp3*	CCGTTCCCTGCTGGAAAGAC	AGGCAACACTCGTCGAAGAC
Insulin-like peptide 7	*Dilp7*	CCTGGCTGCACGTGAACTAT	TGGATGGACAATACTCGGCG
Keren	*Krn*	CCGCTTTAATCGGCGCTTAC	GGTCTCGGCGTTGTGGATAT
Mitf	*Mitf*	AGTATCGGAGTAGATGTGCCAC	CGCTGAGATATTGCCTCACTTG
NADPH oxidase	*Nox*	ACCCATCAACCAGCAGTCAG	GTGCATCCAAATCGAGTACGG
Niemann-Pick type C-2c	*Npc2c*	TCCCTGTGCCTAGTGCTTTCT	GGCTGAGGATAGTTGCTGTCG
puckered	*puc*	GCCACATCAGAACATCAAGC	CCGTTTTCCGTGCATCTT
Ribosomal protein L32	*RpL32*	CGGATCGATATGCTAAGCTGT	CGACGCACTCTGTTGTCG
short gastrulation	*sog*	GAGCGAACCAATCACTCACTC	CAAGTTCTCATTGCTGCCAC
spatzle	*spz*	GTGATTCTGGAAAATGGGATTC	TCTGTGGTGGGTGAAACTTCT
spitz	*spi*	TGCGGTGAAGATAGCCGATC	TTCGCATCGCTGTCCCATAA
Suppressor of cytokine signaling at 36E	*Socs36E*	ATGGGTCATCACCTTAGCAAGT	TCCAGGCTGATCGTCTCTACT
unpaired 1	*upd1*	TGCAGTTGCCGTTCTAGTCA	GCGTGGCGAATAATACTTTCC
unpaired 2	*upd2*	CGGAACATCACGATGAGCGAAT	TCGGCAGGAACTTGTACTCG
unpaired 3	*upd3*	GCAAGAAACGCCAAAGGA	CTTGTCCGCATTGGTGGT
vein	*vn*	TCACACATTTAGTGGTGGAAGC	CGTGACCTCTGCGTTCTGT
wingless	*wg*	CAGGGACGCAAGCATAATAGA	CTGCCGCAGGTTCTTCTC

Table S3: qPCR amplification program

Step	Temperature (°C)	Time (seconds)	Repeats of Cycles
Initial Denaturation	95	30	1
Denaturation	95	10	
Annealing	60	30	40
Extension	65	30	
Final extension	65	60	1

Table S4: 16S colony PCR amplification program

Step	Temperature (°C)	Time	Repeats of Cycles
Initial Denaturation	95	5 min	1
Denaturation	95	20 sec	
Annealing	60-75	30 sec	25
Extension	72	90 sec	
Final extension	72	5 min	1

REFERENCES

Apidianakis, Y.; Pitsouli, C.; Perrimon, N.; Rahme, L. Synergy between bacterial infection and genetic predisposition in intestinal dysplasia. *Proceedings of the National Academy of Sciences* **2009**, *106*, 20883-20888, doi:10.1073/pnas.0911797106.

Apidianakis, Y.; Pitsouli, C.; Perrimon, N.; Rahme, L. Synergy between bacterial infection and genetic predisposition in intestinal dysplasia. *Proceedings of the National Academy of Sciences* **2009**, *106*, 20883-20888, doi:10.1073/pnas.0911797106.

Leader, D.P.; Krause, S.A.; Pandit, A.; Davies, S.A.; Dow, J.A T. FlyAtlas 2: a new version of the Drosophila melanogaster expression atlas with RNA-Seq, miRNA-Seq and sex-specific data. *Nucleic Acids Research* **2017**, *46*, D809-D815, doi:10.1093/nar/gkx976.

Micchelli, C.A.; Perrimon, N. Evidence that stem cells reside in the adult Drosophila midgut epithelium. *Nature* **2006**, *439*, 475-479, doi:10.1038/nature04371.

Neophytou, C.; Pitsouli, C. Biotin controls intestinal stem cell mitosis and host-microbiome interactions. *Cell Rep* **2022**, *38*, 110505, doi:10.1016/j.celrep.2022.110505.

Pitsouli, C.; Delidakis, C. The interplay between DSL proteins and ubiquitin ligases in Notch signaling. *Development* **2005**, *132*, 4041-4050, doi:10.1242/dev.01979.

Tamamouna, V.; Panagi, M.; Theophanous, A.; Demosthenous, M.; Michail, M.; Papadopoulou, M.; Teloni, S.; Pitsouli, C.; Apidianakis, Y. Evidence of two types of balance between stem cell mitosis and enterocyte nucleus growth in the Drosophila midgut. *Development* **2020**, *147*, dev189472, doi:10.1242/dev.189472.

Zeng, X.; Hou, S.X. Enteroendocrine cells are generated from stem cells through a distinct progenitor in the adult Drosophila posterior midgut. *Development* **2015**, *142*, 644-653, doi:10.1242/dev.113357.

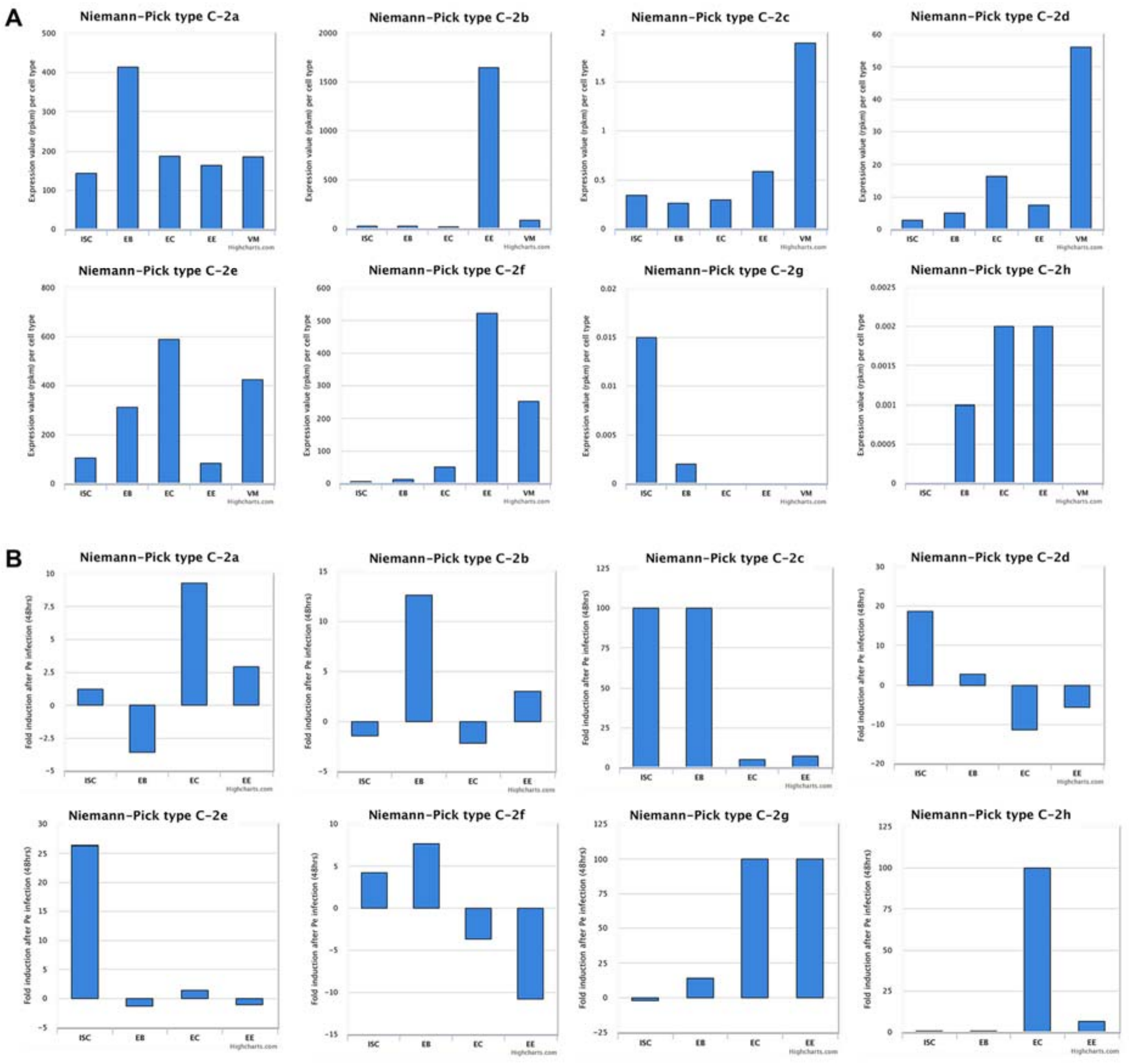

Figure S1. *Npc2* gene family expression in adult midguts. (**A**) *Npc2a-h* transcript expression in different adult midgut cell types. (**B**) *Npc2a-h* transcript expression in different adult midgut cell types upon *P. entomophila* infection. Data retrieved from FlygutSeq (http://flygutseq.buchonlab.com/).

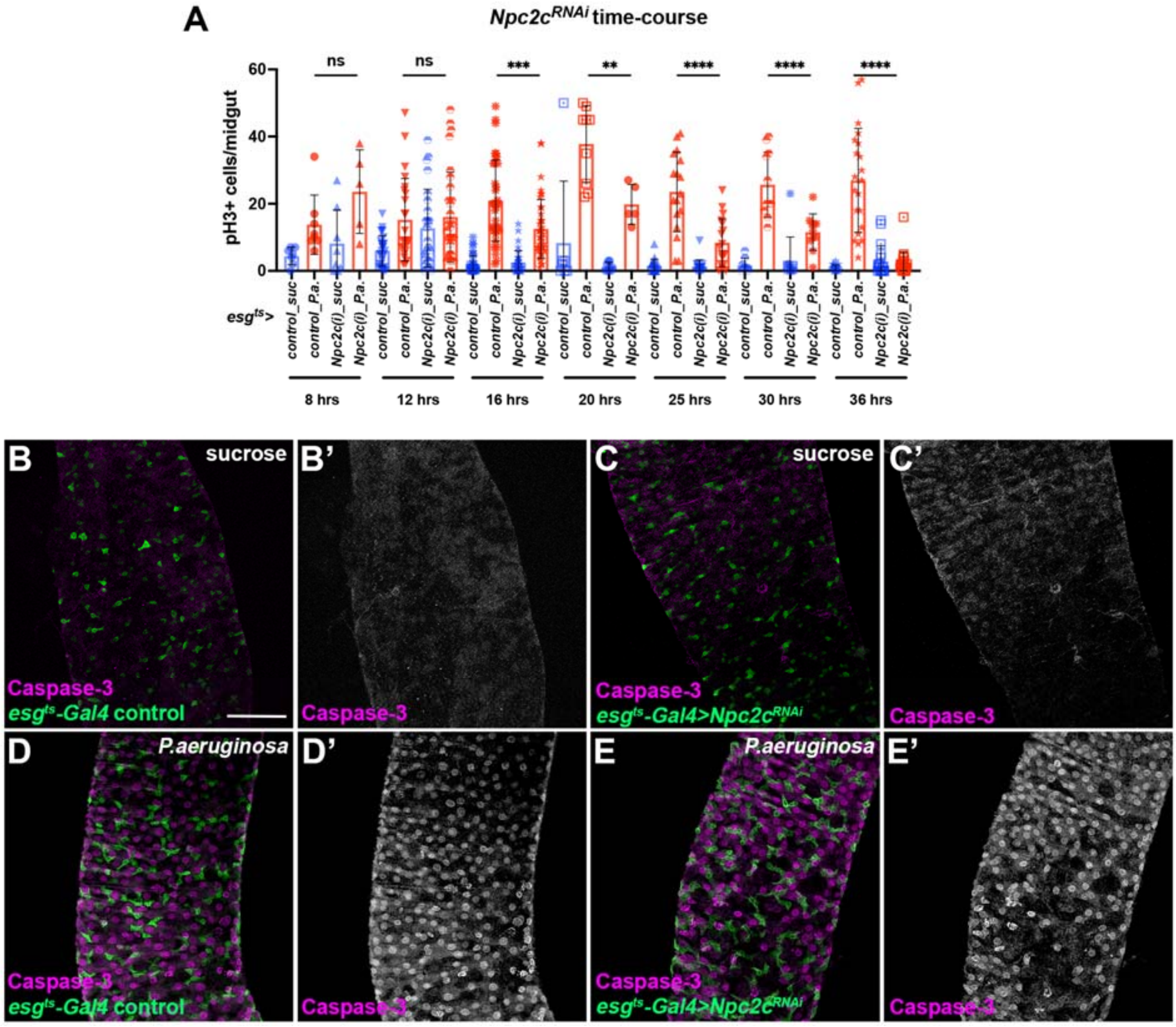

Figure S2. Progenitor-specific *Npc2c* silencing reduces mitosis effectively, but it does not cause apoptosis. (**A**) Quantification of the mitotic index (pH3-positive cells) in *esg^{ts}-Gal4* control and *esg^{ts}-Gal4>Npc2c^{RNAi1}* adult midguts during a time-course experiment of 8, 12, 18, 20, 25, 30, and 36 hrs post-transgene induction in uninfected (blue bars) and *P.a.*-infected (red bars) flies. Mitosis is gradually reduced and by 36 hrs it is eliminated in both uninfected and *P.a.*-infected midguts. (**B-E**) *esg^{ts}-Gal4* control (B,D) and *esg^{ts}-Gal4>Npc2c^{RNAi1}* (C,E) adult posterior midguts without (B-C) and with *P.a.* infection (D-E) stained for anti-cleaved Caspase-3 (magenta). GFP (green) labels the intestinal progenitors. The Caspase-3 channel is shown separately in grey in B'-E'. Although infection induces apoptosis, this is restricted to mature intestinal cells. Scale bars 75 µm. Experiments were repeated at least twice. Statistical significance in A was tested with the Student's t-test: ns, not significant, * $0.01<p\leq0.05$, ** $0.001<p\leq0.01$, *** $0.0001<p\leq0.001$, **** $p\leq0.0001$.

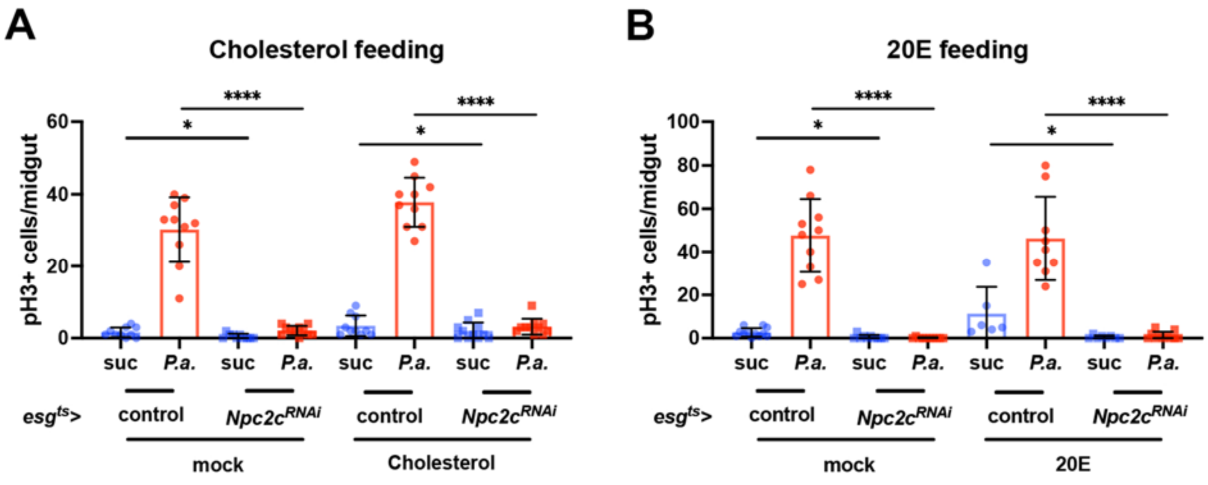

Figure S3. Supplementation of the diet with cholesterol or 20E does not rescue reduced mitosis caused by *Npc2c* silencing. (**A**) Quantification of the mitotic index (pH3-positive cells) in *esg^ts^-Gal4* control and *esg^ts^-Gal4>Npc2c^RNAi1^* uninfected and *P.a.*-infected adult midguts upon cholesterol supplementation. (**B**) Quantification of the mitotic index (pH3-positive cells) in *esg^ts^-Gal4* control and *esg^ts^-Gal4>Npc2c^RNAi1^* uninfected and *P.a.*-infected adult midguts upon 20E supplementation. Error bars correspond to the standard deviation. Experiments were repeated at least twice. Statistical significance in E was tested with the Student's t-test: ns, not significant, * 0.01<p≤0.05, ** 0.001<p≤0.01, *** 0.0001<p≤0.001, **** p≤0.0001.
